# The cost of delivering COVID-19 vaccines in Vietnam

**DOI:** 10.1186/s12913-024-11202-w

**Published:** 2024-07-08

**Authors:** Van Minh Nguyen, Flavia Moi, Laura Boonstoppel, Hong Thi Duong, Chien Chinh Vien, Minh Van Hoang

**Affiliations:** 1 Hanoi University of Public Health, Hanoi, Vietnam; 2 ThinkWell, Geneva, Switzerland; 3 National Institute of Hygiene and Epidemiology, Hanoi, Vietnam; 4 Tay Nguyen Institute of Hygiene and Epidemiology, Dak Lak, Vietnam

**Keywords:** COVID-19, Cost analysis, Bottom-up, Retrospective, Vietnam

## Abstract

**Background:**

The COVID-19 pandemic affected hundreds of millions of people and lives, and vaccination was the safest and most effective strategy to prevent and mitigate the burden of this disease. The implementation of COVID-19 vaccination in Vietnam in 2021 was unprecedentedly challenging in scale and complexity, yet economic evidence on the cost of delivery vaccines thought the program was lacking.

**Methods:**

This retrospective costing study utilized a bottom-up, ingredient-based approach to estimate the cost of delivering COVID-19 vaccines in Vietnam in 2021, from a payer perspective. The study included 38 study sites across all administrative and implementation level, including three geographic areas and two delivery strategies, in two provinces, Hanoi and Dak Lak. The study findings were complemented with qualitative interviews with health staff and stakeholders.

**Results:**

The economic cost to deliver one COVID-19 vaccine dose was $1.73, mostly comprised of opportunity costs ($1.14 per dose) which were driven by labor costs ($1.12 per dose). The delivery cost in urban areas was the highest ($2.02), followed by peri-urban areas ($1.45) and remote areas ($1.37). Delivery costs were higher at temporary sites ($1.78) when compared to facility-based delivery ($1.63). Comparing low-volume and high-volume periods showed that the delivery cost decreased significantly as volume increased, from $5.24 per dose to $1.65 per dose.

**Conclusions:**

The study estimates the cost of delivering COVID-19 vaccines in Vietnam in 2021. Enabling factors and challenges during the implementation of the program were explored. Study limitations may lead to underestimation of results and reduce generalizability.

**Supplementary Information:**

The online version contains supplementary material available at 10.1186/s12913-024-11202-w.

## Background

COVID-19 (C19), an infectious disease caused by a coronavirus known as SARS-CoV-2, was declared a Public Health Emergency of International Concern on January 30th, 2020 [1], kickstarting global concern regarding the outbreak. By August 2023, the World Health Organization (WHO) reported more than 760 million cases of C19, along with 6.9 million deaths worldwide since the start of the pandemic in December 2019 [2]. C19 vaccines have been recommended as the safest and most effective strategy to prevent and protect people from hospitalization or death caused by C19 [3, 4].

Realizing the importance of vaccination, on March 8th, 2021, a national C19 immunization program was launched in Vietnam, prioritizing the most vulnerable populations, due to the constraints in vaccine supply at the time. The initial targeted populations included frontline and essential workers, who were most likely to be exposed to the virus, and elderly and chronic disease patients, who were most at risk of experiencing severe effects from the infection. After four months of implementation targeting prioritized groups, as vaccine supply ramped up, a nationwide, large-scale vaccination program was initiated in July 2021, expanding the target population to all adults aged 18 and over. The goal set out by the program was to vaccinate 75 million people – approximately 76% of the total population in Vietnam – with at least two doses, meaning that 150 million doses had to be delivered by early 2022. In October 2021, the target population was once again expanded to those aged 12 years old or older. The WHO’s global goal of vaccinating at least 70% of each country population by mid-2022 was accomplished by Vietnam by the end of 2021 after only nine months of rollout [5]. The country also exceeded its vaccination goals by delivering over 152 million doses [5].

The C19 vaccination program in Vietnam leveraged the existing management and implementation structure and resources of the National Expanded Program on Immunization (NEPI). This program has been managing routine vaccinations throughout the country since 1981, and its central office is housed within the National Institute of Hygiene and Epidemiology (NIHE). The hierarchy structure includes the Ministry of Health and NEPI central office at national level, NEPI regional offices, provincial Center for Disease Control (CDC), district health centers (DHC) and commune health centers (CHC).

The implementation of C19 vaccination program in Vietnam was organized in rounds, with each round corresponding to the arrival of one vaccine lot in the country. The duration of each round varied, ranging from 1 to 15 days, depending on the number of vaccine doses received and allocated that round. During the high-volume period (from July to December 2021), as vaccine shipments became more frequent, vaccination rounds could overlap, with new ones starting while a previous round was still ongoing. Upon the arrival of vaccine lots, the NEPI would collect and inspect the vaccines and then distribute them down to lower administrative levels, including regional offices and provincial CDCs.

Two vaccination strategies were deployed to deliver C19 vaccines in Vietnam, facility-based and delivery at temporary sites. Facility-based immunization sites were deployed at district or commune health centers, hospitals, vaccination clinics, general health clinics, and other health facilities that qualified to provide the service. Temporary sites, on the other hand, were set up to handle larger delivery volumes during high-volume periods, or in some cases, during low-volume periods when the commune health center did not have sufficient space to administer C19 vaccines in accordance with the C19 prevention recommendations. To accommodate a large number of people while respecting social distancing regulations, temporary sites were usually set up at schools, large office buildings, industrial areas, stadiums, or community centers. Vaccination teams deployed at immunization sites were comprised on one vaccinator and other support staff responsible for welcoming beneficiaries, screening them for pre-existing conditions and providing a brief consultation on the benefits and potential adverse effects of vaccination, record-keeping, crowd control, and post-vaccination monitoring. The number of vaccination team members varied across immunization sites and was based on the availability of health workers. Vaccination teams at temporary sites were staffed from local health facilities, district and commune health centers.

The delivery of C19 vaccines posed unprecedented challenges to Vietnam’s public health system, in terms of program scale and complexity. Meanwhile, delivery costs were highly uncertain, as at the time of conducting this study there were only two publications on the cost of delivering C19 vaccines and neither was based on actual costs incurred. A modelling effort published in 2021 by the COVAX Working Group on Delivery Cost projected delivery cost across 92 low- and middle-income countries to be US$ 1.41 per dose, or US$ 3.15 per person vaccinated with two doses, including wastage [6]. While these modeled estimates were valuable to inform budgeting and planning decisions at the time, they had significant limitations due to assumptions on the delivery strategy mix, recruitment of additional health staff, and payment of financial incentives to health workers. Another study [7] estimated the incremental cost of introducing C19 vaccines in Kenya with different coverage levels (30%, 50%, 70% and 100%) and found the economic delivery costs to range between $7.17 to $12.22 per person vaccinated with two doses (2021 US dollar, respectively for 100% and 30% coverage). While it provided important evidence for Kenyan policy-makers, this study was based on expenditure plans rather than actual costs [7]. 

To address the evidence gap on actual delivery costs incurred, this study estimated the cost of delivering C19 vaccines in Vietnam, including through different delivery strategies, in different geographic areas, and at different periods of the program in 2021.

## Methods

### Study design

This was a retrospective costing study of the C19 vaccination program in Vietnam in 2021. The study estimated the full financial and economic cost of delivering the vaccines to the targeted populations, included costs related to planning, managing, implementing, and reporting on the program. We excluded the costs of vaccines, due to the sensitivity around these data. The study utilized a payer perspective, estimating all costs incurred at all levels of the public health system in Vietnam, including contributions from key development partners in Vietnam (WHO and UNICEF). The study included administrative and management costs at national, regional, provincial, district, and commune level, and implementation costs at vaccine delivery sites.

In this study, financial costs were defined as financial outlays incurred in 2021 that were specifically related to the C19 vaccination program in Vietnam, such as procurement of immunization supplies and new cold chain equipment for the C19 vaccination program, financial incentives for C19 vaccination team members, salary for newly hired program personnel, etc. Opportunity costs were defined as the value of using a share of existing resources for the C19 vaccination program (see Table 1 for allocation rules used for shared resources). Opportunity costs were monetized based on available information and existing guidance [8]. For paid labor, the fully loaded salary per minute worked (including fringe and benefits) was estimated based on annual salary for each staff, and then multiplied by the number of minutes worked on the C19 vaccination program by that staff, while volunteer labor was monetized multiplying the hours worked by volunteers by the monthly minimum wage. Use of existing cold chain equipment and vehicles was valued based on the purchasing year, the discounted original acquisition price (or current market value when acquisition price was not available), and annualized following Vietnam’s Ministry of Finance regulation on equipment useful life. For all other shared costs—including fuel, vehicle and cold chain equipment maintenance, electricity, printing—written records on total funds allocated to each site for these resource were available. The economic cost presented in this study was the sum of financial and opportunity cost, representing the total value of all resources used.

**Table 1:** Allocation rules for shared costs

Resource type	Allocation rule
Paid labor	Paid labor costs were allocated to the C19 vaccination program based on a self-reported timesheet indicating how much time each staff spent on C19 vaccination program activities.
Fuel and vehicle maintenance	Fuel and vehicle maintenance costs incurred for all programs were allocated to the C19 vaccination program based on the share of vehicles that were used for C19 vaccination activities.
Electricity	Total electricity costs were allocated to the C19 program based on the share of square meters occupied by cold storage facilities (assuming that cold storage was exclusively used for C19 vaccines during the study period), and by program days proportion in 2021.
Printing	Shared printing costs at each site were allocated using the proportion of staff annual working time dedicated to C19 vaccination program activities.
CCE and CCE maintenance	Proportion of the C19 vaccination program duration in 2021.

*Abbreviations: CCE: cold chain equipment; C19: COVID-19

The study applied a bottom-up (or ingredients-based) costing approach, to estimate the costs by resource type and program activity. The program activities were based on a generic research protocol for C19 vaccine delivery costing studies [9] which was adapted to the Vietnamese context following the Ministry of Health’s COVID-19 vaccination plan, and informed by consultations with vaccination program experts from the NEPI managers at the national level. The full definitions of the program activities and resource types included in this study can be found in Supplementary Table S1 and Table S2.

The study estimated the delivery costs for an initial low-volume period and a high-volume period of the vaccination program in Vietnam in 2021. The low-volume period was defined as the first rounds of the C19 vaccination, from March to June 2021, when the supply of vaccines was low, and the target population was limited to prioritized populations. The high-volume period from July to December 2021 was characterized by a surge in the delivery of C19 vaccines, due to the increased vaccine supply, which allowed the country to expand the eligibility criteria to all adults aged 18 and above.

All cost results presented are in 2021 US dollars (USD) and amounts collected in Vietnamese Dong (VND) were converted using a rate of 1 USD = 23,145 VND (the official exchange rate of the State Bank of Vietnam on 30/12/2021 [10]). For capital items, straight-line depreciation was applied to newly acquired equipment based on their useful life (as set by the Vietnamese Ministry of Finance), while for existing capital items, opportunity capital costs were calculated based on annualization with a 3% discount rate [11]. Where necessary, values were converted to 2021 price using the Consumer Price Index published by the Vietnam’s General Office of Statistics [12].

Complementary in-depth qualitative interviews with government officials and health staff at study sites were conducted to understand the implementation of the program and explore any operational and financial challenges the health system in Vietnam may have faced during the roll out of the program.

### Sampling

A four-stage purposive sampling approach was used to select a total of 38 study sites, which included 26 vaccination sites and 12 administrative and management sites. Table 2 provides more details on study sites’ contributions to the C19 vaccination program, while an overview of the sample provided in Table 3.

**Table 2:** Description of study sites involved in the C19 vaccination program

Entity	Contributions to the C19 vaccination program in 2021
NEPI central and regional offices	Technical consultation, overall program management (e.g., development and implementation of guidelines on training, supervision, reporting), vaccine procurement and distribution, and vaccine quality control.
Development partners (WHO, UNICEF)	Technical and financial support in developing guidelines, supervision, national-level training, implementation-level social mobilization and advocacy. Financial donations to support microplanning, social mobilization and service delivery. In-kind donations including vaccines, immunization supplies, and cold chain equipment.
Provincial CDCs	Managing the implementation of C19 vaccinations at all districts within the province; receiving vaccines from the NEPI national or regional offices, storing and delivering vaccines to lower administrative levels.
District health centers	Managing the implementation of C19 vaccinations at all communes within the district; receiving vaccines from the provincial CDCs, storing and delivering vaccines to commune health centers as well as administering C19 vaccines at the district health centers’ general clinics.
Commune health centers	Microplanning, storing and administering vaccines, and reporting on vaccine delivery performance.
Immunization sites	Providing immunization services to the eligible population.

In collaboration with the NEPI managers at the national level, the research team purposively selected Hanoi province, a predominantly urban area, and Dak Lak, a remote province. Then, a total of six districts were selected, two urban and two peri-urban districts in Hanoi and two remote districts in Dak Lak. From each district in Hanoi, two communes were chosen, and in Dak Lak’s province districts, one commune was selected per district. All immunization sites within the sampled communes were included in the study. This study also included the two main development partner organizations, WHO and UNICEF.

**Table 3:** Study sample

Level		Number of sites			Total
		Urban	Peri-urban	Remote	
**Administrative level**	National/Regional offices	4			4
	Provincial CDCs	2			2
	District health centers	2	2	2	6
**Subtotal for administrative level study sites**					**12**
**Implementation level**	Commune health centers	4	6	2	10
	*Facility-based immunization sites*	5	8	1	14
	*Temporary immunization sites*	4	6	2	12
**Subtotal for implementation level study sites**					**26**
**Grand total**					**38**

### Data collection

The cost data was collected through standardized questionnaires, administered in Microsoft Excel spreadsheets. Cost data collection was done in-person by Hanoi University of Public Health research team members. The process was conducted between April to May 2022 for vaccination sites and government entities, and between August to September 2022 for the development partners. The data collected related to C19 vaccination program were mainly from the study sites’ financial records, inventory records and staff interviews.

### Data analysis

The cost of delivering C19 vaccines was estimated for each of the study sites, by calculating and allocating the costs to each of the program activities and each of the resource types. The average cost per dose at each of the study sites were calculated by dividing the total cost incurred by the total number of vaccine doses delivered at that site. Then, the total delivery cost per dose for each implementation site was obtained by aggregating vertically: the cost per dose for the vaccination site was added together with the cost per dose for the commune, district, province, and region in which the vaccination site was located, as well as the national level cost per dose. The volume-weighted average delivery cost per dose across all study immunization sites was then estimated with the bootstrap method, as our sample size was small and not random [13]. The variance of the mean cost per dose was calculated based on the formula for the variance of a ratio [13], as the cost per dose is equivalent to the ratio between total cost and total volume delivered. The following formula was used to estimate the variance:

$$V \left(\raisebox{1ex}{$X$}\!\left/ \!\raisebox{-1ex}{$Y$}\right.\right)=E\left(\raisebox{1ex}{${X}^{2}$}\!\left/ \!\raisebox{-1ex}{${Y}^{2}$}\right.\right)-{\left[E\left(\raisebox{1ex}{$X$}\!\left/ \!\raisebox{-1ex}{$Y$}\right.\right)\right]}^{2}$$

Where X represents the total cost, Y represents the total volume, E is the expected value (mean), and V represents the variance. The 95% confidence intervals were calculated for all estimates but were not reported for all findings as they were too narrow. The bootstrapping was performed in STATA 17 using the *bsample* package [14].

### Scenario analysis

A scenario analysis was conducted to estimate the delivery costs associated with three alternative financial incentive schemes. Our in-depth interviews with health staff and stakeholders revealed that the compensation for health workers who participated in the C19 vaccination program was considered to be inadequate. According to the scheme, each vaccination team member was meant to receive $0.32 per dose delivered. However, there was a daily cap of $6.48, which corresponds to a maximum of 20 doses per day per staff, which is far less than what staff delivered on average, especially during the high-volume period. In addition, only vaccination team members received compensation, while microplanning and social mobilization staff did not.

Based on input from vaccination experts from the NEPI and findings from the qualitative interviews, the research team designed three scenarios that outline alternative injection incentive schemes. The three scenarios, described in Table 4, were designed to estimate the economic cost of delivering C19 vaccines if participating staff received a more comprehensive compensation.

**Table 4:** Description of scenarios

Scenario	Vaccination team members	Microplanning and social mobilization staff
**Baseline**	$0.32 per dose per member, capped at $6.48 per member per day (excluding recordkeeping staff)	No financial incentive
**Scenario 1**	$6.48 per member per vaccination day (*including* recordkeeping staff)	$6.48 per member per day of microplanning and social mobilization (1 day/round)
**Scenario 2**	$0.32 per dose per member, no cap (*including* recordkeeping staff)	No financial incentive
**Scenario 3**	$0.32 per dose per member, no cap (*including* recordkeeping staff)	$6.48 per member per day of micro planning and social mobilization (1 day/round)

## Results

### Characteristics of sampled delivery sites

A total of 26 delivery sites were included in this study. On average, each delivery site had 30 vaccination team members, including 9 regular staff and 21 additional staff (Table 5). Temporary sites had higher daily delivery volume, 446 doses per day, compared to facility-based sites, with 247 doses per day.

**Table 5:** Characteristics of delivery sites

	Volume weighted average (rounded)						
	Overall	Facility-Based	Temporary sites	Hanoi			Dak Lak
				Overall	Urban	Peri-Urban	Overall/ Remote
Number of sites	26	14	12	23	9	14	3
Vaccination team members per site	30	17	43	30	31	29	25
Vaccinators per site	7	2	13	8	7	9	2
Person-minute spent to deliver one dose (*both periods*)	28	15	42	29	35	22	22
Doses delivered per day (*both periods*)	396	247	446	391	389	393	345
Doses delivered per day per site *(low-volume period)*	98	101	84	99	111	90	32
Doses delivered per day per site *(high-volume period)*	412	272	468	417	414	421	347

Vaccine delivery was more labor intensive at temporary sites, as this strategy was mainly utilized during the high-volume periods to maximize the delivery capacity, which was not possible at facility-based sites. Larger waiting and implementation areas were required to accommodate more vaccine recipients but also to comply with the social distancing regulations, hence, on average, temporary sites within our sample reported having more vaccination team members (47 members compared to 17 at facility-based sites), more vaccinators deployed at each site (an average of 13 vaccinators at temporary sites versus 2 vaccinators at facility-based sites), and higher person-minutes per dose (temporary sites needed 42 person-minute of labor per dose compared to 15 person-minute per dose at facility-based sites). Detailed delivery sites’ characteristics for the low- and high-volume periods can be found in Supplementary Table S6 & Table S7.

### Overall delivery cost per dose

**Fig. 1:**
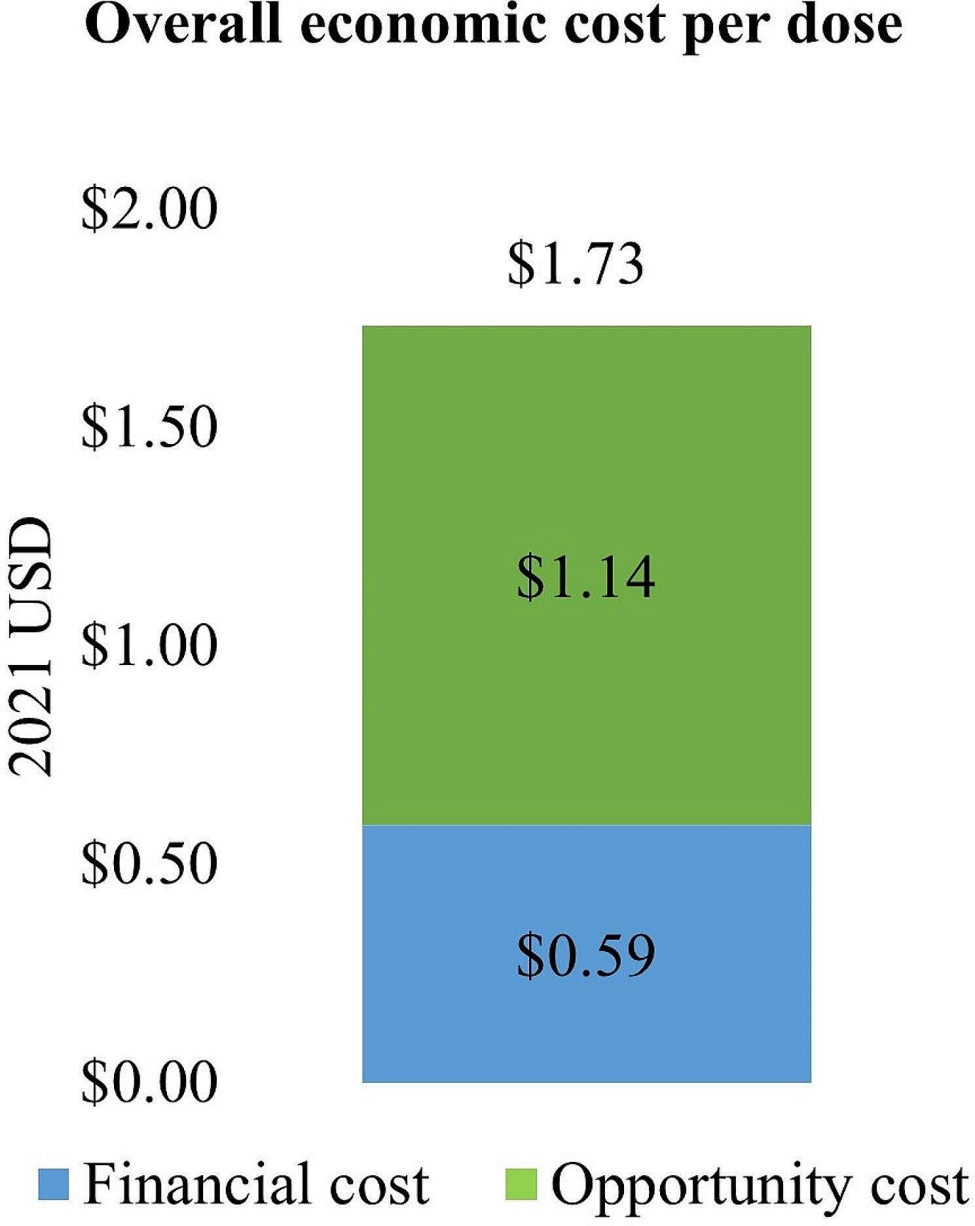
Overall economic cost per dose

Our study estimated that the average economic cost per dose for delivering C19 vaccines in Vietnam in 2021 was $1.73. Financial costs made up 34% of that ($0.59) and opportunity cost accounted for 66% of the total economic cost ($1.14) (Fig. 1).

**Fig. 2:**
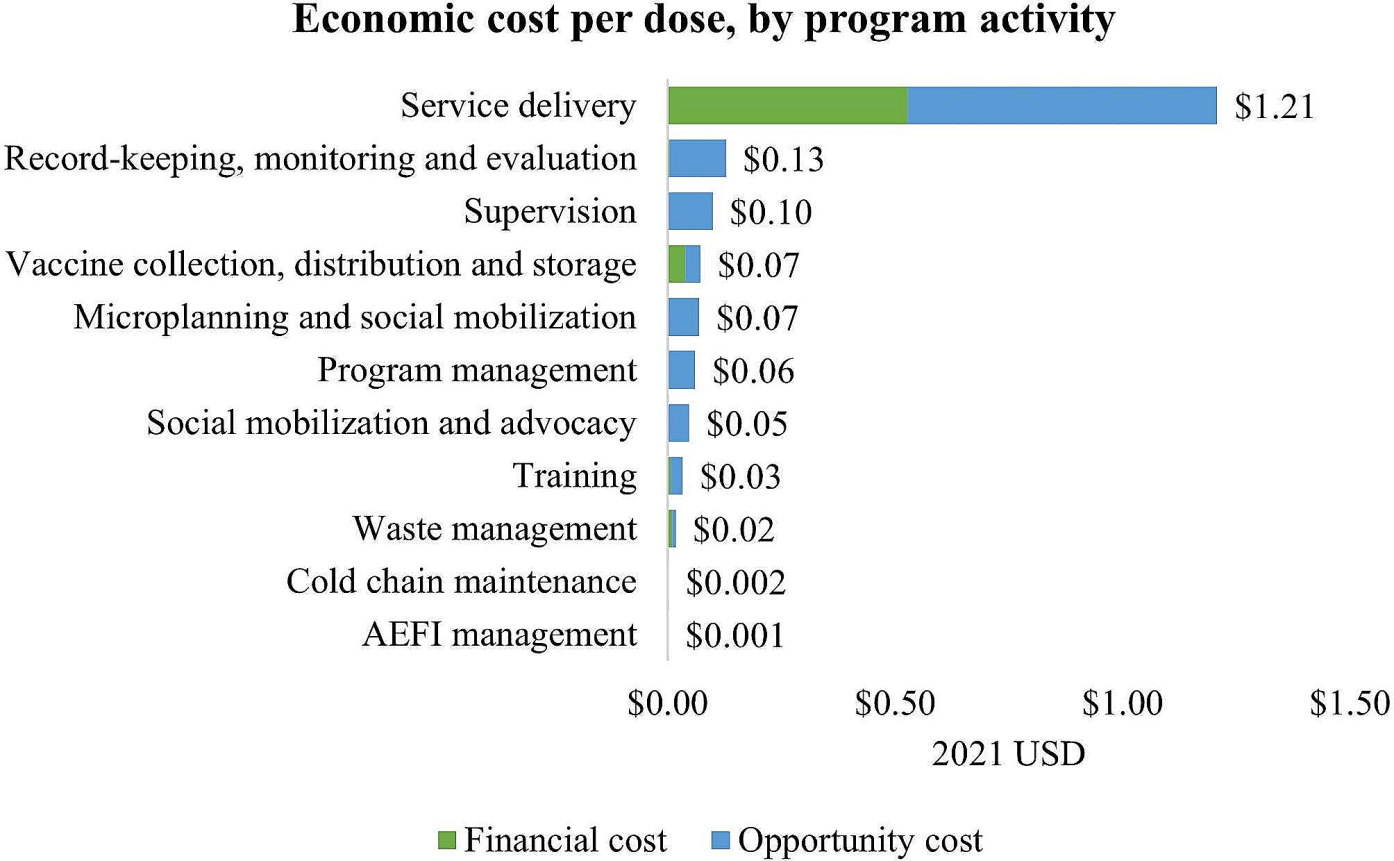
Economic cost per dose, by program activity

Figure 2 shows the economic cost per dose of delivering a dose of C19 vaccine by program activity. Service delivery was the most expensive component of the vaccination, estimated at $1.21 per dose. Other program activities that were cost drivers included record-keeping, monitoring and evaluation, supervision, and vaccine collection, distribution and storage (See Supplementary Table S3 for details).

**Fig. 3:**
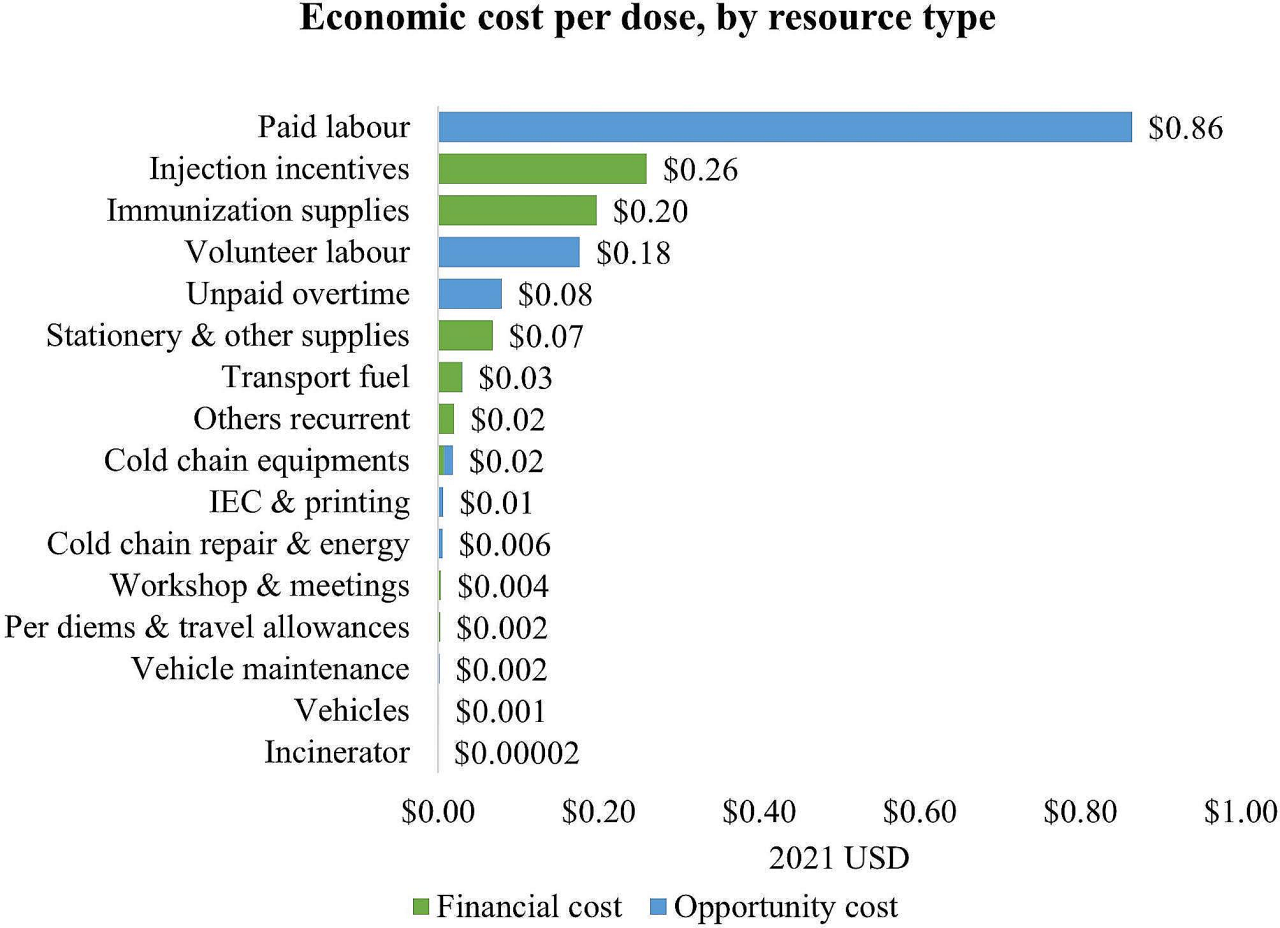
Economic cost per dose, by resource type

Figure 3 shows that resource types that related to service delivery—such as labor, injection incentives or immunization supplies—were the largest drivers of the C19 vaccine delivery cost. Paid labor was fully comprised of opportunity costs, reflecting the value of leveraging the existing workforce to implement the C19 vaccination program. No financial resources were available to hire additional health workers, and the program exclusively relied on its existing health workforce and on volunteers. The limited injection incentives given to vaccination team members and vaccine injection and safety supplies were the main financial costs incurred for the vaccination program (See Supplementary Table S3 for details).

### Cost differences between delivery strategies, vaccination periods, and geographic locations

**Fig. 4:**
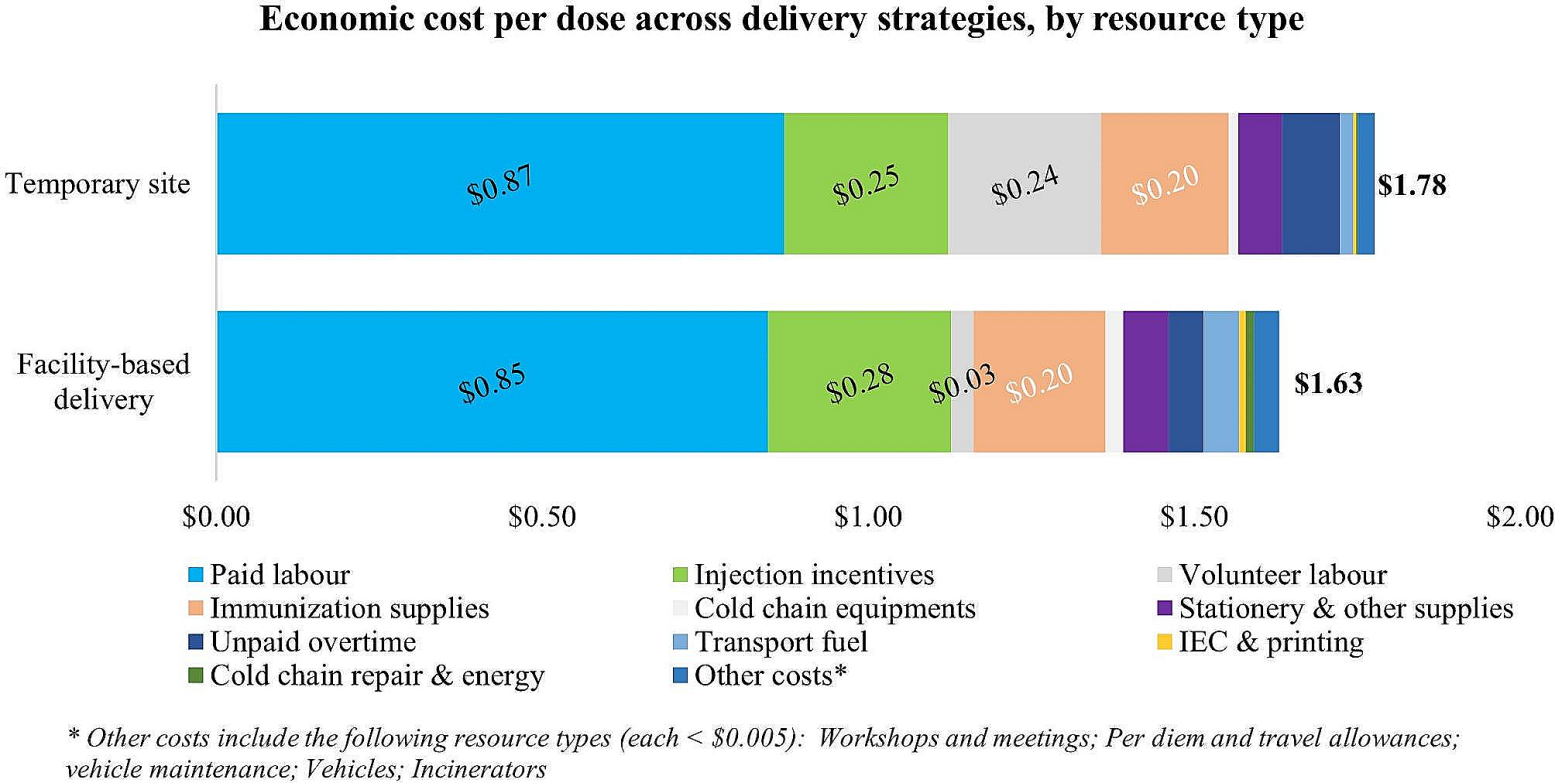
Economic cost per dose across delivery strategies, by resource type

The cost per C19 dose delivered was $1.63 for facility-based delivery, and $1.78 for delivery at temporary sites. The delivery cost at temporary sites was slightly higher at facility-based sites due to the higher volunteer labor costs (Fig. 4) (See Supplementary Table S3 for details).

**Fig. 5:**
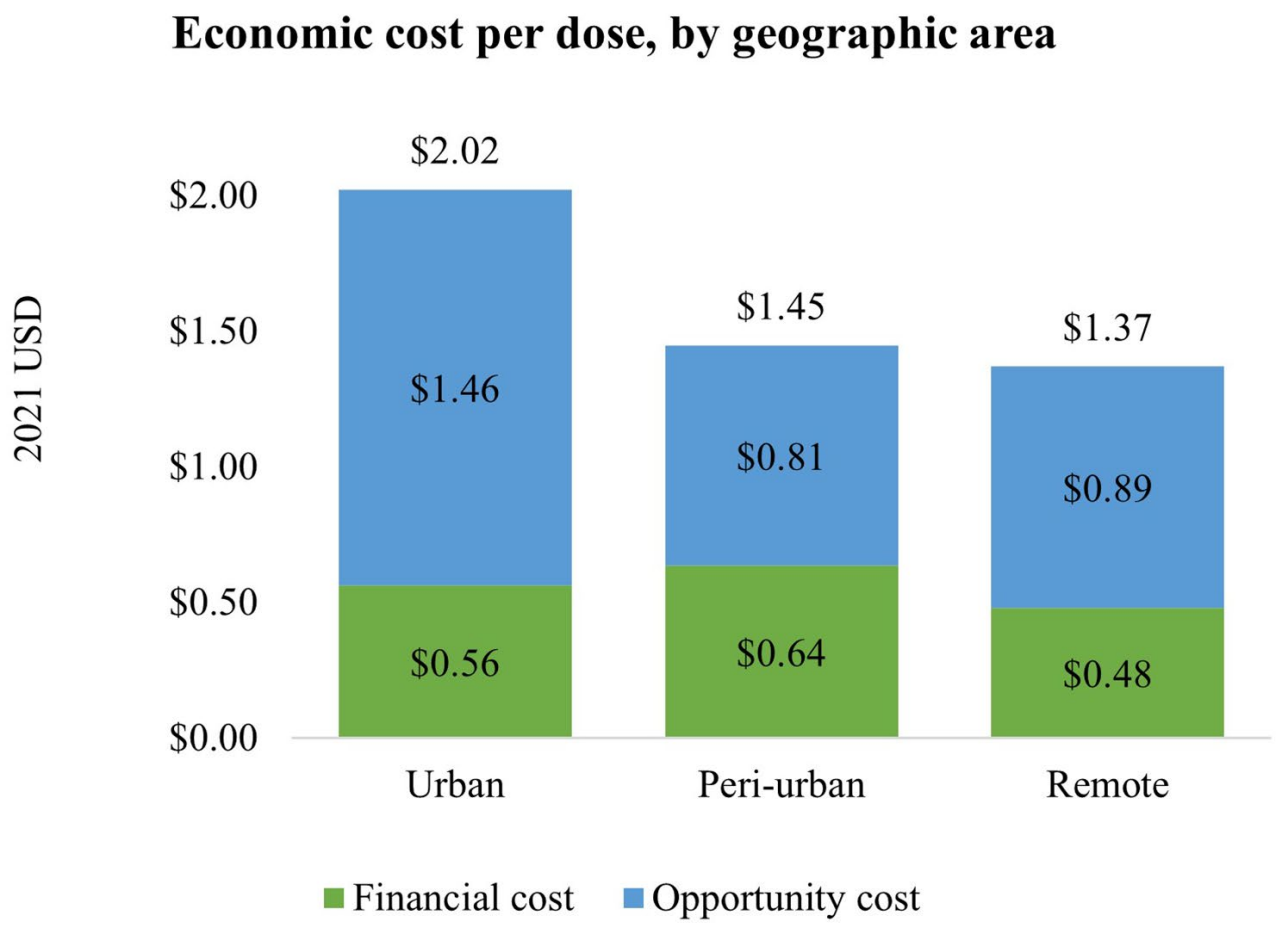
Economic cost per dose, by geographic area

The delivery costs differed by geographic area. Vaccination sites in urban areas had the highest delivery cost per dose ($2.02) and highest delivery volume on average per implementation site (27,270 doses/site), followed by peri-urban sites ($1.45 per dose and 16,299 doses per site) and remote areas ($1.37 per dose and 10,025 doses per site) (Fig. 5). Urban areas had the highest paid labor costs, at $1.09/dose, compared to peri-urban ($0.62/dose) and remote area ($0.74/dose) (See Supplementary Table S4 and Figure S2 for details).

**Fig. 6:**
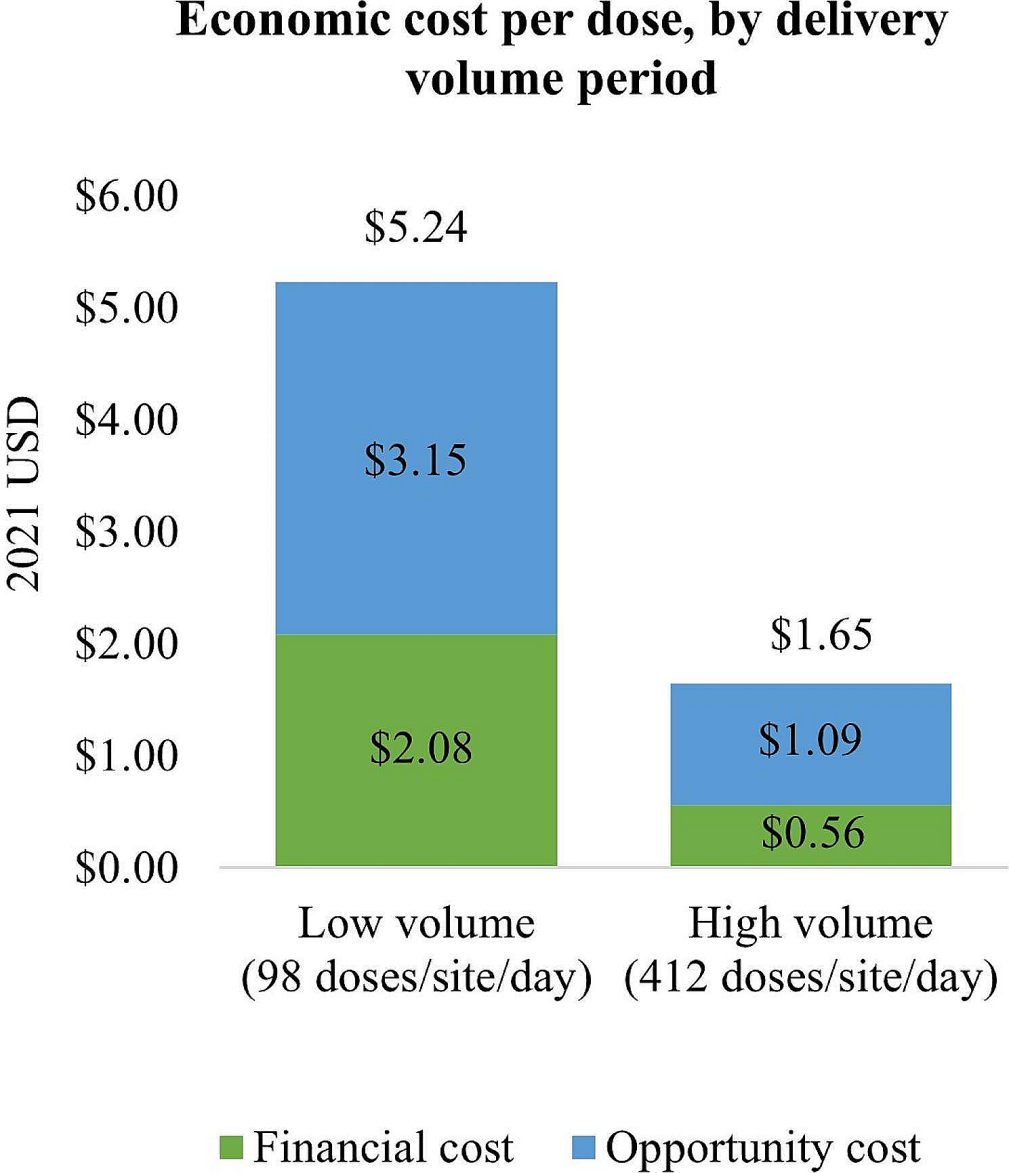
Economic cost per dose, by delivery volume period

A sharp decrease in financial cost and economic cost per dose delivered was observed as the vaccination program shifted from the low-volume period (from March to June 2021 with the average daily delivery volume of 98 doses/site) to the high-volume period (July to December 2021, on average 412 doses delivered per site per day), as shown in Fig. 6. The average economic cost per dose delivered during the low-volume period was $5.24, while the cost for the high-volume period was $1.65 per dose delivered. The higher delivery cost during the low-volume period was largely driven by paid labor and injection incentives costs. The unit cost of injection incentives, vaccine transportation, distribution and storage all decreased significantly when the program scaled up (See Supplementary Table S5 and Figure S1 for details).

### Scenario analysis

**Fig. 7:**
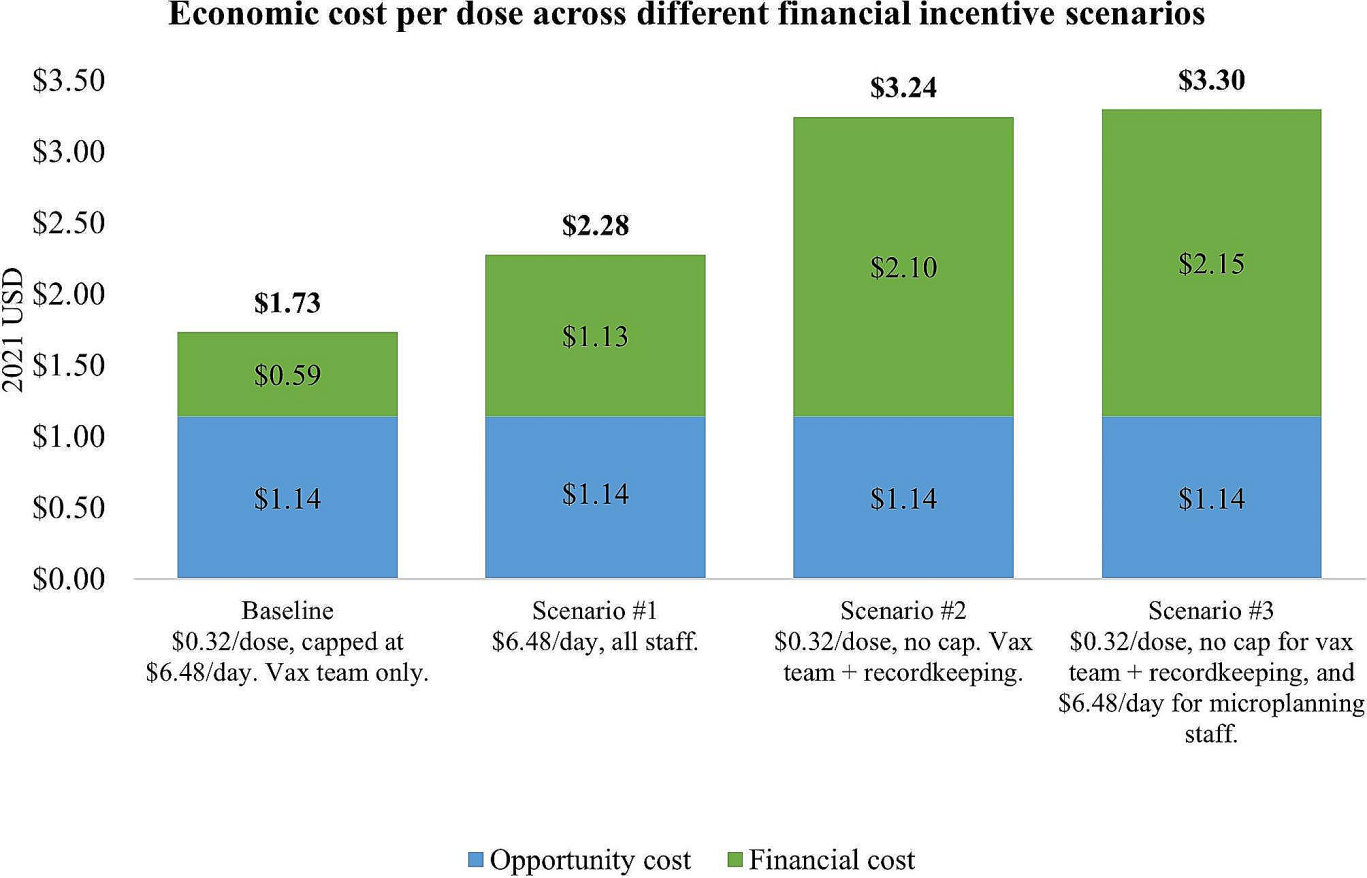
Economic cost per dose across different financial incentives scenarios

Vaccinators’ financial incentives were capped at $6.48 per day. During interviews, vaccination team members expressed that they considered these incentives highly inadequate given the long working days (10 to 12 hours per day) which extended for a long period of time. Moreover, key members of the vaccination team were excluded from the incentive scheme. Expanding the existing financial incentive scheme to include microplanning and recordkeeping staff would bring the financial cost per dose to $1.13, a 32% increase compared to the baseline. Only adding recordkeeping staff while removing the daily cap would bring the financial cost per dose to $2.10 (87% increase). Finally, expanding the scheme to compensate microplanning and recordkeeping staff while also removing the daily cap for vaccination team members and recordkeeping staff would bring the financial cost per dose to $2.15 (91% increase) (Fig. 7).

### Qualitative findings: enabling factors and challenges

Several enabling factors were uncovered through in-depth qualitative interviews with staff from the study sites. First, effective collaboration and staff commitment were the essential ingredient for the program’s success. Multisectoral collaboration was an important characteristic of the program as both the health sector and outside of the health sector entities pooled and utilized all available resources. Within health sector, public and private healthcare facilities and medical universities mobilized all human resources to support the delivery of vaccination. Outside of health sector, several entities filled resource gaps as needed: private airline companies and the military forces supported the transportation of vaccines across the country; Ministry of Information and Technology, Ministry of National Defense and technology organizations developed C19 mobile applications to track and manage vaccination records; military bases supported vaccine storage and distribution. Second, despite social distancing regulations, necessary trainings for health staff and social mobilization were delivered promptly for the effective and safe rollout of C19 vaccines. Vietnam leveraged technology platforms (such as Zoom) to provide trainings to health staff at all levels of administration and implementation, reducing burden and costs of organizing and attending trainings, which were plentiful, as new vaccine products were rolled out and delivery protocols were regularly updated to ensure the best and safest practices were implemented. Furthermore, instant message mobile application (e.g. Zalo, a popular mobile application in Vietnam, similar to WhatsApp or Viber) was utilized to identify and mobilize the target population for each round of vaccination. The third enabling factor for the C19 program in Vietnam was that lockdowns facilitated social mobilization and microplanning and increased the number of available volunteers. During the surge waves of C19, Vietnam implemented lockdowns to slow the spread of the virus. These lockdowns facilitated microplanning and social mobilization as many lines of work were suspended and residents stayed at home. Consequently, the target populations were easier to reach, and more individuals were available for volunteering to support the vaccination program. Lastly, Vietnam achieved high vaccine coverage by promptly tackling initial vaccine hesitancy. During the first weeks of the program’s implementation, many in the target population had reservations in accepting the vaccine, due to concerns related to vaccines’ safety and efficacy, and doubts on the need of getting vaccinated. This initial hesitancy was quickly and effectively addressed through a heightened focus on social mobilization and the production and distribution of tailored advocacy material at implementation level.

Despite enabling factors that led to the success of the C19 vaccination program, health workers in Vietnam faced and managed numerous challenges. Firstly, health workers were overburdened due to high workload, high pressure to deliver vaccines quickly, and staff shortages. During the high-volume period, many health workers reported working 10 to 12 hours per day regularly, with peaks of up to 16 hours per day and nearly no time off. The workload of the health workers was caused by long service delivery sessions, repeated training, and laborious reporting and verification processes. This period also overlapped with a surge of C19 spread driven by the Delta variant, underscoring the need for a successful vaccination program, and intensifying the significant stress on the health workers. Secondly, the unpredictability and high frequency of vaccination rounds led to inefficiencies in planning and vaccine distribution. The arrival of vaccine lots in the country would trigger a series of preparation activities that had to be completed within a few days, such as planning and coordinating among all entities involved and levels of management and implementation to ensure a smooth rollout. However, the exact timing of the arrival was mostly unknown due to variability in shipping and importing procedures. This led to significant overtime for staff across roles and administrative levels. Thirdly, insufficient cold chain equipment further burdened overloaded health workers. Due to the lack of cold chain capacity and ultra-cold chain equipment at lower levels, health workers were pressured to distribute the vaccines across the system and deliver vaccines to recipients quickly. Shortage of adequate transporting vehicles and cold boxes also added the additional workload to health workers, as they had to do multiple vaccine collection trips for the same shipment of vaccines, sometimes multiple trips were made in a same day, particularly when the vaccine volume was large (e.g. for Sinopharm vaccines).

## Discussion

This study assessed the cost of delivering C19 vaccines to target populations in Vietnam during 2021. From the payer perspective, the economic cost to deliver a vaccine dose was $1.73 on average, which was primarily driven by the opportunity cost of existing health workers’ time. The economic cost per dose is low compared with other vaccine delivery costing studies conducted in Vietnam. A 2008–2010 study of the economic cost of HPV vaccine delivery estimated that it would cost $2.75 per dose to vaccinate 10-year-olds girls in Vietnam at a facility-based site, or $2.98 per dose for school-based sites [15]. 

The average financial cost of delivering C19 vaccines found in this study ($0.59 per dose) was primarily driven by injection incentives offered to vaccinators. The financial cost was low compared to a 2017 study which estimated the fiscal cost of delivering a tetanus toxoid (TT) and tetanus-diphtheria (Td) vaccine dose to childbearing age women (15–39 years old) in Vietnam to be between $2.17 to $2.33 (in 2021 USD), depending on the proportion of doses delivered through facilities versus schools [16]. 

A modelling exercise conducted by the COVAX Working Group on Delivery Costs projected the financial delivery cost in Vietnam to range between $0.73 and $1.85 per C19 dose, which is higher than the financial cost per dose found in our study [17]. The lowest COVAX estimate assumed 15% of the C19 doses would be delivered through outreach, while the higher estimate assumed half of all doses would be delivered through outreach. The model assumed that additional outreach activities would drive up financial expenditures primarily for health workers’ per diems. In reality, Vietnam delivered many more doses through temporary sites than projected by COVAX, but as the incentives provided to health workers were very limited, this did not result in additional financial expenditures. The COVAX model also assumed that a significant share of health workers would be recruited for the C19 vaccination effort, resulting in additional financial expenses, but we found no additional hiring at any of our sampled sites.

Few studies of the cost of delivering C19 vaccines in low- or middle-income country settings have been published. This study was part of a multi-country study in seven low and middle-income countries, which followed a similar standardized methodology [9]. The unit cost of delivery in Vietnam was lower than that in Côte d’Ivoire [18], the Philippines [19], and Uganda, but higher than the cost found in Bangladesh and Mozambique [20]. A study conducted in Kenya projected the incremental cost of introducing C19 vaccines to range from $7.17 to $12.22 per person vaccinated with two doses (2021 US dollar), and in Botswana a fiscal cost of $17.95 and an economic cost of $31.15 per dose was found (2021 US dollar), much higher than the findings of our study [7, 21]. 

This study found that the economic cost of delivery at temporary sites ($1.78 per dose) was higher than at facility-based sites ($1.63 per dose), while the financial cost of deliver was slightly lower at temporary sites ($0.56) compared with facility-based sites ($0.66). The latter is an unusual finding, as non-facility based delivery is generally more costly, as was the case in the TT/Td costing study where the fiscal cost of outreach was higher than that for facility-based delivery [16]. This is explained by the very low financial resources available for the Vietnamese C19 vaccination program for per diems and transport, which are typically cost drivers of outreach and temporary site delivery.

We observed a sharp decrease in the economic unit cost of delivery from $5.25 during the first low-volume months of the program to $1.65 per dose C19 delivered during the latter half of 2021 which was characterized by high delivery volumes. This demonstrates the economies of scale the C19 vaccination program was able to achieve, as each vaccinator was able to immunize more people within a same vaccination session or more vaccine vials were transported per trip. Furthermore, financial injection incentives, one of the drivers of the financial cost per dose, also reduced significantly as the delivery volume surged, as they were capped at a fixed amount per vaccinator per day. Given the fact that the delivery volume of the C19 vaccination program has reduced considerably since 2021, finding other ways to contain costs at lower delivery volumes will be important to ensure cost-efficient delivery of reaching smaller target populations.

The low unit cost of delivery found in our study must not be interpreted as a reflection of low resource needs, but rather of the limited availability of financial resources to support the roll-out of a vaccination program that was unprecedented in terms of scale and complexity. The C19 vaccine program demanded the delivery of extremely high volumes in a short period of time, which stretched the Vietnamese health system to its limits. This caused disruptions to the delivery of other health services, which is a cost to the health system that is not reflected in the unit cost of delivering C19 vaccines estimated in this study.

The compensation scheme for health workers, in the form of injection incentives, was considered insufficient in magnitude and did not cover all staff involved (only vaccinators). Our scenario analysis estimated that providing incentives to all staff would have increased the economic cost per dose by 32%, or even by 87% if the scheme was not capped at a fixed amount per day. The C19 vaccination program could rely on an exceptional level of commitment from the Vietnamese health workforce given the unprecedented crisis brought by the pandemic, but as not all vaccination programs may be able to do so in the future, adequate financial compensation for all health workers must be budgeted for in the case of large immunization campaigns held in the future.

Our study has several limitations. First, the cost estimates were drawn from a small sample size. In total, the study included two provinces, out of 63 provinces in the country, and from the two provinces, only six districts were chosen. The small sample size of 38 study sites including 26 vaccination sites limits the generalizability of our findings. Second, the research team was not able to include costs from other government organizations, such as the military forces. Based on the interviews at national and regional levels, we acknowledged the contributions of the Vietnamese military bases in transportation, distribution, and storage, which could be up to 50% of all C19 vaccine doses delivered during high-volume period in 2021 across the country. However, relevant information to estimate or impute the magnitude of support was confidential and could not be made available to the research team. This limitation means that vaccine transport costs will have been underestimated in our study, though this would not have changed the findings on what were the key cost drivers for C19 vaccine delivery.

## Conclusion

The study findings provided financial and economic cost estimates for the delivery of C19 vaccines to evolving target populations in Vietnam in 2021. The study demonstrated that considerable economies of scale were achieved when supply constraints were lifted and vaccination opened up to general population, suggesting that this rapid roll-out strategy may have been a very efficient and effective way to vaccinate the target population quickly during an emergency period. However, given the considerable burden this posed on the existing health workforce, and the stretched health systems infrastructure, this disrupted the delivery of other health services, and would not have been sustainable in the longer term.

### Electronic supplementary material

Below is the link to the electronic supplementary material.

Supplementary Material 1

## Data Availability

The data supporting this article is publicly available through the Harvard Dataverse at https://dataverse.harvard.edu/dataset.xhtml? persistentId=doi:10.7910/DVN/V1PWYV.
