# Supplementary material for: The cost of delivering COVID-19 vaccines in Vietnam
Source: BMC Health Serv Res. 2024 Jul 8;24:779. doi: 10.1186/s12913-024-11202-w (PMC11232236; doi:10.1186/s12913-024-11202-w)

# Annexes

## Annex 1: Program activities and resource types definitions

Table S1. Program activities definitions

| Program activity | Definition |
| --- | --- |
| **Program management** | C19 vaccination program management, including: development of guidelines, program meetings, development of vaccination implementation plan for each round, budgeting for the program. |
| **Vaccine collection, distribution and storage** | Vaccine acquisition procedures; Vaccine collection at the airports or other distribution points, storing vaccines in national or subnational cold stores, distributing vaccines down to the facility, and to outreach or program sites where relevant. |
| **Cold chain maintenance** | Maintaining and repairing the cold chain for the purpose of the C19 vaccine roll-out. |
| **Training** | Attending and/or providing C19 vaccination-related training, including topics such as administering vaccines, storage and logistics, record keeping, pharmacovigilance, social mobilization, planning, supervision, etc. |
| **Social mobilization and advocacy** | Mainly advocacy activities, such as: developing and distributing advocating materials, via mass media, social media and leaflets. |
| **Supervision** | Supervising subordinate or peer health or community workers. |
| **Service delivery: facility-based delivery** | Including the administration of the vaccine to people within the district general clinics and commune health centres, preparation and cleaning up before and after the vaccination event. |
| **Service delivery: temporary sites** | Including Traveling to and from temporary sites outside of the facility, the act of administering the vaccine and supporting vaccine administration (crowd control, screening, setting up and cleaning up the vaccination site before and after). |
| **Waste management** | Time and resources spent on disposing sharps and infectious non-sharp wastes. |
| **AEFI management** | Managing and following up on post-vaccination events following C19 vaccine administration; Developing reports on AEFI events occurred. |
| **Record-keeping, HMIS, monitoring and evaluation** | Data entry and analysis, reporting, monitoring. |
| **Microplanning and social mobilization** | Referring to the development of eligible participants lists for each round and inviting eligible participants coming in vaccination sites in the area. |

Table S2. Resource types definitions

| Resource types | Description | Financial vs. opportunity cost | Start-up vs. operating | Data source |
| --- | --- | --- | --- | --- |
| **Recurrent costs** | | | |  |
| **Paid labor** | Paid salary for health staff and government officers. The paid personnel costs were derived from the total working time of each staff and their annual salary in 2021. | Opportunity cost | Operating, unless related to start-up activities | Annual salaries including fringe and benefits from each study site’s financial records.  Self-reported timesheet with estimation of total time spent on each program activity, collected at all study sites at all levels (implementation and administrative). |
|  | Paid salary for new staff that were hired specifically for C19 vaccination program. Based on the financial records of the study site(s). | Financial cost | Operating, unless related to start-up activities |  |
| **Volunteer labor** | Value of volunteer labor (medical students, local youth members, etc.) for those staff who are not receiving salary from the government/MOH. This cost was calculated based on each volunteer’s working time and valued at minimum wage (specific for the region of each study site). | Opportunity cost | Operating, unless related to start-up activities | Monthly minimum wage for each region, according to Decree 145/2020/NĐ-CP.  Self-reported timesheet with estimation of total time spent on each program activity, collected at all study sites at all levels (implementation and administrative). |
| **Unpaid overtime** | Unpaid overtime of health staff related to C19 vaccination activities, defined as any time worked in excess of the regular working time during the study period (calculated based on a 6-day work week, an 8-hour workday and 11 days of holiday in 2021). | Opportunity cost | Operating, unless related to start-up activities | Same as paid labor |
| **Per diem and travel allowances** | Per diem and travel allowances paid to regular staff as well as volunteers for participation to activities related to the C19 vaccination program. | Financial cost | Operating | Total per diem and travel allowances were collected from the COVID-19 financial records at the study site at all levels (implementation and administrative). |
| **Injection incentives** | Performance-based injection incentives, of the value of 7,500 VND per delivered dose and per each vaccination team member, capped at 150,000VND per member, per day (which corresponds to 20 doses per member per day). The average injection incentive per dose may vary from site to site due to the capped amount paid per day to each vaccination team member. | Financial cost | Operating | Total injection incentives paid (or approved budget that may have not fully paid yet) were collected at all study sites at all levels (implementation and administrative). |
| **Vaccine injections and safety supplies** | Cost for immunization supplies and personal protective equipment. | Financial cost | Operating | The total costs for immunization supplies, personal protective equipment was collected at all study site at all levels (implementation and administrative). |
| **Stationery and other supplies** | Cost for stationery and IEC materials required for the program. | Financial cost | Operating | The total costs for stationery and IEC materials were collected at all study site at all levels (implementation and administrative). |
| **Transport and fuel** | Fuel costs specifically for C19 vaccination program activities that required travelling (supervision, trainings, vaccine collection, distribution, etc.) | Financial cost | Operating | Total fuel cost was collected from the COVID-19 financial records at all study site at all levels (implementation and administrative). |
|  | A proportion of total cost for gasoline at the study site which was used for C19 vaccination program activities. | Opportunity cost | Operating | Total fuel cost from the financial records at all study sites at all levels (implementation and administrative). |
| **Vehicle maintenance** | Cost for vehicles maintenance specifically done for C19 vaccination program in 2021. | Financial cost | Start-up | Total vehicle maintenance costs were collected from the COVID-19 financial records of study site at all levels (implementation and administrative). |
|  | Routine and non-routine vehicle maintenance done in 2021. | Opportunity cost | Operating | Total vehicle maintenance costs were collected at study sites at all levels (implementation and administrative). |
| **Cold chain equipment repairs and energy costs** | Cost for CCE maintenance specifically done for C19 vaccination program in 2021. | Financial cost | Start-up | The maintenance cost for CCE was collected from the COVID-19 financial records at all study sites at all levels (implementation and administrative). |
|  | Routine and non-routine cold chain maintenance/repairs done in 2021  The energy cost for the CCE is the energy bill of the storage room (if available) | Opportunity cost | Operating | Total electricity costs were collected at all study sites at all levels (implementation and administrative). |
| **Printing cost** | Share of the site’s printing for 2021 spent in relation to C19 vaccination activities | Opportunity cost | Operating, unless related to start-up activities | Total printing costs were collected at all study sites at all levels (implementation and administrative). |
|  | Cost incurred specifically for C19 vaccination program as reported in financial reports (if available), or estimations based on number of pages printed per each participant (per dose delivered) at implementation sites. | Financial cost | Operating | The printing cost was collected from the COVID-19 financial records at all study sites at all levels (implementation and administrative). |
| **Workshops and meetings** | Cost incurred specifically for C19 vaccination workshops and meetings (line of budget, if available) | Financial cost | Start-up | The total cost of trainings and workshops was collected from the COVID-19 financial records at all study sites at all levels (implementation and administrative). |
|  | Cost incurred for general workshops and meetings which was also used for C19 vaccination program | Opportunity cost | Start-up | Total cost of trainings and workshops were collected at all study sites at all levels (implementation and administrative). |
| **Waste disposal fuel** | Costs for fuel used in incinerators for C19 vaccination program specifically. | Financial cost | Operating | COVID-19 and/or regular financial and inventory records from the study sites. |
|  | Share of routine waste disposal incinerator fuel costs that was used in relation to C19 vaccine waste management. | Opportunity cost | Operating | Total cost for waste disposal fuel at all study sites at all levels (implementation and administrative).  Total waste disposal volume and C19 vaccination waste disposal volume. |
| **Other recurrent cost** | Other financial outlays that are not included in the categories above, including direct financial support for development of guidelines and policies and vaccine acquisition costs at National level, waste disposal (if carried out by a third party) at district level, sugar drinks for vaccine recipients, etc. | Financial cost | Operating, unless related to start-up activities | The total costs for all other expenditures that were directly used for C19 vaccination were collected at all study sites at all levels (implementation and administrative). |
| **Capital costs** | | | |  |
| **Cold chain equipment** | Depreciation costs of existing cold chain equipment used for C19 vaccine storage at study sites | Opportunity cost | Operating | Acquisition cost, quantity, number of useful life years and number of years used were collected for all existing CCE at all study sites (implementation and administrative). |
|  | New cold chain equipment acquired in 2021 and used for C19 vaccination program. | Financial cost | Start-up | The total cost of newly acquired equipment and used for C19 vaccination program was collected at all study sites at all levels (implementation and administrative). |
| **Vehicles** | Depreciation costs of existing vehicle(s) used for C19 vaccination activities (trainings, supervision, vaccine collection/distribution) at study sites | Opportunity cost | Operating | Acquisition cost, quantity, number of useful life years and number of years used were collected for the vehicles that were used for C19 vaccination program at all study sites (implementation and administrative). |
|  | New vehicle(s) acquired in 2021 and used for C19 vaccination program. | Financial cost | Start-up | The total cost of newly acquired vehicles and used for C19 vaccination program was collected at all study sites at all levels (implementation and administrative). |
| **Incinerators** | Depreciation costs of existing incinerator(s) used for C19 vaccination waste disposal at study sites | Opportunity cost | Operating | Acquisition cost, quantity, number of useful life years and number of years used were collected for the incinerators that were used for C19 vaccination program at all study sites (implementation and administrative). |
|  | New incinerator(s) acquired in 2021 and used for C19 vaccination program. | Financial cost | Start-up | The total cost of newly acquired incinerators and used for C19 vaccination program was collected at all study sites at all levels (implementation and administrative). |

Annex 2: Detailed cost findings

Table S3. Financial, opportunity and economic cost per dose, by delivery strategy and time period, for program activities and resource types

|  |  | **Overall** | | | **Delivery strategy** | | | | | | **Time period** | | | | | |
| --- | --- | --- | --- | --- | --- | --- | --- | --- | --- | --- | --- | --- | --- | --- | --- | --- |
|  |  |  |  |  | **Facility-based** | | | **Temporary sites** | | | **Low volume** | | | **High-volume** | | |
|  | **Note: True zero values in grey* | **Fin** | **Opp** | **Eco** | **Fin** | **Opp** | **Eco** | **Fin** | **Opp** | **Eco** | **Fin** | **Opp** | **Eco** | **Fin** | **Opp** | **Eco** |
| **Program activities** | Program management | 0.00 | 0.06 | 0.06 | 0.00 | 0.05 | 0.05 | 0.00 | 0.06 | 0.06 | 0.02 | 0.31 | 0.33 | 0.00 | 0.05 | 0.05 |
|  | Vaccine collection, distribution and storage | 0.04 | 0.03 | 0.07 | 0.07 | 0.04 | 0.11 | 0.02 | 0.03 | 0.05 | 0.77 | 0.43 | 1.20 | 0.02 | 0.02 | 0.04 |
|  | Cold chain maintenance | 0.00 | 0.00 | 0.00 | 0.00 | 0.00 | 0.00 | 0.00 | 0.00 | 0.00 | 0.00 | 0.02 | 0.02 | 0.00 | 0.00 | 0.00 |
|  | Training | 0.01 | 0.03 | 0.03 | 0.01 | 0.05 | 0.06 | 0.01 | 0.01 | 0.02 | 0.06 | 0.24 | 0.31 | 0.00 | 0.02 | 0.03 |
|  | Social mobilization and advocacy | 0.00 | 0.05 | 0.05 | 0.00 | 0.07 | 0.07 | 0.00 | 0.03 | 0.04 | 0.00 | 0.68 | 0.68 | 0.00 | 0.03 | 0.03 |
|  | Supervision | 0.00 | 0.10 | 0.10 | 0.00 | 0.11 | 0.11 | 0.00 | 0.09 | 0.09 | 0.01 | 0.40 | 0.41 | 0.00 | 0.09 | 0.09 |
|  | Service delivery | 0.53 | 0.68 | 1.21 | 0.55 | 0.58 | 1.13 | 0.52 | 0.72 | 1.24 | 1.02 | 0.88 | 1.90 | 0.52 | 0.67 | 1.20 |
|  | Record-keeping, monitoring and evaluation | 0.00 | 0.12 | 0.13 | 0.01 | 0.04 | 0.05 | 0.00 | 0.16 | 0.16 | 0.07 | 0.12 | 0.19 | 0.00 | 0.12 | 0.12 |
|  | Waste management | 0.01 | 0.01 | 0.02 | 0.01 | 0.01 | 0.03 | 0.01 | 0.00 | 0.01 | 0.11 | 0.03 | 0.14 | 0.01 | 0.01 | 0.01 |
|  | AEFI management | 0.00 | 0.00 | 0.00 | 0.00 | 0.00 | 0.00 | 0.00 | 0.00 | 0.00 | 0.00 | 0.00 | 0.00 | 0.00 | 0.00 | 0.00 |
|  | Microplanning and social mobilization | 0.00 | 0.07 | 0.07 | 0.00 | 0.00 | 0.00 | 0.00 | 0.09 | 0.10 | 0.00 | 0.03 | 0.03 | 0.00 | 0.07 | 0.07 |
| **Resource types** | Paid labor | 0.00 | 0.86 | 0.86 | 0.00 | 0.84 | 0.85 | 0.00 | 0.87 | 0.87 | 0.01 | 2.68 | 2.69 | 0.00 | 0.81 | 0.81 |
|  | Unpaid overtime | 0.00 | 0.08 | 0.08 | 0.00 | 0.05 | 0.05 | 0.00 | 0.09 | 0.09 | 0.00 | 0.06 | 0.06 | 0.00 | 0.08 | 0.08 |
|  | Volunteer labor | 0.00 | 0.18 | 0.18 | 0.00 | 0.03 | 0.03 | 0.00 | 0.24 | 0.24 | 0.00 | 0.00 | 0.00 | 0.00 | 0.17 | 0.17 |
|  | Per diems & travel allowances | 0.00 | 0.00 | 0.00 | 0.00 | 0.00 | 0.00 | 0.00 | 0.00 | 0.00 | 0.01 | 0.00 | 0.01 | 0.00 | 0.00 | 0.00 |
|  | Injection incentives | 0.26 | 0.00 | 0.26 | 0.28 | 0.00 | 0.28 | 0.25 | 0.00 | 0.25 | 0.75 | 0.00 | 0.75 | 0.25 | 0.00 | 0.25 |
|  | Immunization supplies | 0.20 | 0.00 | 0.20 | 0.20 | 0.00 | 0.20 | 0.20 | 0.00 | 0.20 | 0.20 | 0.00 | 0.20 | 0.20 | 0.00 | 0.20 |
|  | Stationery & other supplies | 0.07 | 0.00 | 0.07 | 0.07 | 0.00 | 0.07 | 0.07 | 0.00 | 0.07 | 0.07 | 0.00 | 0.07 | 0.07 | 0.00 | 0.07 |
|  | Transport fuel | 0.03 | 0.00 | 0.03 | 0.06 | 0.00 | 0.06 | 0.02 | 0.00 | 0.02 | 0.60 | 0.00 | 0.60 | 0.02 | 0.00 | 0.02 |
|  | Vehicle maintenance | 0.00 | 0.00 | 0.00 | 0.00 | 0.00 | 0.00 | 0.00 | 0.00 | 0.00 | 0.00 | 0.00 | 0.00 | 0.00 | 0.00 | 0.00 |
|  | Cold chain repair & energy | 0.00 | 0.01 | 0.01 | 0.00 | 0.01 | 0.01 | 0.00 | 0.00 | 0.00 | 0.00 | 0.13 | 0.13 | 0.00 | 0.00 | 0.00 |
|  | IEC & printing | 0.00 | 0.01 | 0.01 | 0.00 | 0.01 | 0.01 | 0.00 | 0.00 | 0.00 | 0.00 | 0.09 | 0.09 | 0.00 | 0.00 | 0.00 |
|  | Workshop & meetings | 0.00 | 0.00 | 0.00 | 0.00 | 0.00 | 0.00 | 0.00 | 0.00 | 0.00 | 0.03 | 0.00 | 0.03 | 0.00 | 0.00 | 0.00 |
|  | Others recurrent costs | 0.02 | 0.00 | 0.02 | 0.03 | 0.00 | 0.03 | 0.02 | 0.00 | 0.02 | 0.26 | 0.00 | 0.26 | 0.01 | 0.00 | 0.01 |
|  | Cold chain equipment | 0.01 | 0.01 | 0.02 | 0.01 | 0.01 | 0.03 | 0.00 | 0.01 | 0.01 | 0.12 | 0.16 | 0.28 | 0.00 | 0.01 | 0.01 |
|  | Vehicles | 0.00 | 0.00 | 0.00 | 0.00 | 0.00 | 0.00 | 0.00 | 0.00 | 0.00 | 0.00 | 0.03 | 0.03 | 0.00 | 0.00 | 0.00 |
|  | Incinerator | 0.00 | 0.00 | 0.00 | 0.00 | 0.00 | 0.00 | 0.00 | 0.00 | 0.00 | 0.00 | 0.00 | 0.00 | 0.00 | 0.00 | 0.00 |
| **OVERALL** | | **0.59** | **1.14** | **1.73** | **0.66** | **0.97** | **1.63** | **0.56** | **1.22** | **1.78** | **2.06** | **3.15** | **5.22** | **0.56** | **1.09** | **1.65** |

Table S4. Financial, opportunity and economic cost per dose, across provinces and geographic areas, for program activities and resource types

|  |  | **Province** | | | | | | **Geographic area** | | | | | | | | |
| --- | --- | --- | --- | --- | --- | --- | --- | --- | --- | --- | --- | --- | --- | --- | --- | --- |
|  |  | **Hanoi** | | | **Dak Lak** | | | **Urban** | | | **Peri-urban** | | | **Remote** | | |
|  | **Note: True zero values in grey* | **Fin** | **Opp** | **Eco** | **Fin** | **Opp** | **Eco** | **Fin** | **Opp** | **Eco** | **Fin** | **Opp** | **Eco** | **Fin** | **Opp** | **Eco** |
| **Program activities** | Program management | 0.00 | 0.06 | 0.06 | 0.00 | 0.03 | 0.03 | 0.00 | 0.05 | 0.06 | 0.00 | 0.07 | 0.07 | 0.00 | 0.03 | 0.03 |
|  | Vaccine collection, distribution and storage | 0.04 | 0.03 | 0.07 | 0.02 | 0.04 | 0.06 | 0.03 | 0.03 | 0.06 | 0.05 | 0.03 | 0.08 | 0.02 | 0.04 | 0.06 |
|  | Cold chain maintenance | 0.00 | 0.00 | 0.00 | 0.00 | 0.00 | 0.00 | 0.00 | 0.00 | 0.00 | 0.00 | 0.00 | 0.00 | 0.00 | 0.00 | 0.00 |
|  | Training | 0.01 | 0.03 | 0.03 | 0.00 | 0.02 | 0.02 | 0.01 | 0.02 | 0.03 | 0.00 | 0.03 | 0.03 | 0.00 | 0.02 | 0.02 |
|  | Social mobilization and advocacy | 0.00 | 0.05 | 0.05 | 0.00 | 0.00 | 0.01 | 0.00 | 0.08 | 0.08 | 0.00 | 0.02 | 0.02 | 0.00 | 0.00 | 0.01 |
|  | Supervision | 0.00 | 0.10 | 0.10 | 0.01 | 0.03 | 0.04 | 0.00 | 0.17 | 0.17 | 0.00 | 0.02 | 0.02 | 0.01 | 0.03 | 0.04 |
|  | Service delivery | 0.53 | 0.70 | 1.23 | 0.42 | 0.44 | 0.86 | 0.51 | 0.95 | 1.45 | 0.57 | 0.40 | 0.97 | 0.42 | 0.44 | 0.86 |
|  | Record-keeping, monitoring and evaluation | 0.00 | 0.13 | 0.13 | 0.01 | 0.07 | 0.07 | 0.00 | 0.14 | 0.14 | 0.01 | 0.12 | 0.12 | 0.01 | 0.07 | 0.07 |
|  | Waste management | 0.01 | 0.01 | 0.02 | 0.00 | 0.01 | 0.01 | 0.01 | 0.01 | 0.02 | 0.01 | 0.01 | 0.02 | 0.00 | 0.01 | 0.01 |
|  | AEFI management | 0.00 | 0.00 | 0.00 | 0.00 | 0.00 | 0.00 | 0.00 | 0.00 | 0.00 | 0.00 | 0.00 | 0.00 | 0.00 | 0.00 | 0.00 |
|  | Microplanning and social mobilization | 0.00 | 0.06 | 0.06 | 0.01 | 0.25 | 0.26 | 0.00 | 0.01 | 0.01 | 0.00 | 0.11 | 0.11 | 0.01 | 0.25 | 0.26 |
| **Resource types** | Paid labor | 0.00 | 0.87 | 0.87 | 0.00 | 0.74 | 0.74 | 0.00 | 1.08 | 1.09 | 0.62 | 0.62 | 0.62 | 0.00 | 0.74 | 0.74 |
|  | Unpaid overtime | 0.00 | 0.08 | 0.08 | 0.00 | 0.04 | 0.04 | 0.00 | 0.14 | 0.14 | 0.00 | 0.02 | 0.02 | 0.00 | 0.04 | 0.04 |
|  | Volunteer labor | 0.00 | 0.18 | 0.18 | 0.00 | 0.05 | 0.05 | 0.00 | 0.21 | 0.21 | 0.00 | 0.15 | 0.15 | 0.00 | 0.05 | 0.05 |
|  | Per diems & travel allowances | 0.00 | 0.00 | 0.00 | 0.02 | 0.00 | 0.02 | 0.00 | 0.00 | 0.00 | 0.00 | 0.00 | 0.00 | 0.02 | 0.00 | 0.02 |
|  | Injection incentives | 0.27 | 0.00 | 0.27 | 0.13 | 0.00 | 0.13 | 0.25 | 0.00 | 0.25 | 0.29 | 0.00 | 0.29 | 0.13 | 0.00 | 0.13 |
|  | Immunization supplies | 0.20 | 0.00 | 0.20 | 0.19 | 0.00 | 0.19 | 0.19 | 0.00 | 0.19 | 0.00 | 0.00 | 0.21 | 0.19 | 0.00 | 0.19 |
|  | Stationery & other supplies | 0.07 | 0.00 | 0.07 | 0.07 | 0.00 | 0.07 | 0.07 | 0.00 | 0.07 | 0.00 | 0.00 | 0.07 | 0.07 | 0.00 | 0.07 |
|  | Transport fuel | 0.03 | 0.00 | 0.03 | 0.02 | 0.00 | 0.02 | 0.03 | 0.00 | 0.03 | 0.04 | 0.00 | 0.04 | 0.02 | 0.00 | 0.02 |
|  | Vehicle maintenance | 0.00 | 0.00 | 0.00 | 0.00 | 0.02 | 0.02 | 0.00 | 0.00 | 0.00 | 0.00 | 0.00 | 0.00 | 0.00 | 0.02 | 0.02 |
|  | Cold chain repair & energy | 0.00 | 0.01 | 0.01 | 0.00 | 0.01 | 0.01 | 0.00 | 0.01 | 0.01 | 0.00 | 0.00 | 0.00 | 0.00 | 0.01 | 0.01 |
|  | IEC & printing | 0.00 | 0.01 | 0.01 | 0.01 | 0.00 | 0.02 | 0.00 | 0.01 | 0.01 | 0.00 | 0.00 | 0.00 | 0.01 | 0.00 | 0.02 |
|  | Workshop & meetings | 0.00 | 0.00 | 0.00 | 0.00 | 0.00 | 0.00 | 0.01 | 0.00 | 0.01 | 0.00 | 0.00 | 0.00 | 0.00 | 0.00 | 0.00 |
|  | Others recurrent costs | 0.02 | 0.00 | 0.02 | 0.03 | 0.00 | 0.03 | 0.02 | 0.00 | 0.02 | 0.02 | 0.00 | 0.02 | 0.03 | 0.00 | 0.03 |
|  | Cold chain equipment | 0.01 | 0.01 | 0.02 | 0.00 | 0.01 | 0.01 | 0.00 | 0.01 | 0.01 | 0.03 | 0.02 | 0.03 | 0.00 | 0.01 | 0.01 |
|  | Vehicles | 0.00 | 0.00 | 0.00 | 0.00 | 0.00 | 0.00 | 0.00 | 0.00 | 0.00 | 0.00 | 0.00 | 0.00 | 0.00 | 0.00 | 0.00 |
|  | Incinerator | 0.00 | 0.00 | 0.00 | 0.00 | 0.00 | 0.00 | 0.00 | 0.00 | 0.00 | 0.00 | 0.00 | 0.00 | 0.00 | 0.00 | 0.00 |
| **OVERALL** | | **0.60** | **1.16** | **1.76** | **0.48** | **0.89** | **1.37** | **0.56** | **1.46** | **2.02** | **0.64** | **0.81** | **1.45** | **0.48** | **0.89** | **1.37** |

Table S5. Financial, opportunity and economic cost per dose, across time periods and delivery strategies, for program activities and resource types

|  |  | **Low-volume period** | | | | | | **High-volume period** | | | | | |
| --- | --- | --- | --- | --- | --- | --- | --- | --- | --- | --- | --- | --- | --- |
|  |  | **Facility-based** | | | **Temporary** | | | **Facility-based** | | | **Temporary** | | |
|  | **Note: True zero values in grey* | **Fin** | **Opp** | **Eco** | **Fin** | **Opp** | **Eco** | **Fin** | **Opp** | **Eco** | **Fin** | **Opp** | **Eco** |
| **Program activities** | Program management | 0.02 | 0.31 | 0.33 | 0.02 | 0.32 | 0.34 | 0.00 | 0.03 | 0.03 | 0.00 | 0.06 | 0.06 |
|  | Vaccine collection, distribution and storage | 0.79 | 0.40 | 1.19 | 0.68 | 0.55 | 1.23 | 0.02 | 0.01 | 0.03 | 0.02 | 0.03 | 0.05 |
|  | Cold chain maintenance | 0.00 | 0.02 | 0.02 | 0.00 | 0.01 | 0.01 | 0.00 | 0.00 | 0.00 | 0.00 | 0.00 | 0.00 |
|  | Training | 0.06 | 0.28 | 0.33 | 0.09 | 0.09 | 0.18 | 0.00 | 0.04 | 0.04 | 0.01 | 0.01 | 0.02 |
|  | Social mobilization and advocacy | 0.00 | 0.73 | 0.73 | 0.00 | 0.42 | 0.42 | 0.00 | 0.02 | 0.03 | 0.00 | 0.03 | 0.03 |
|  | Supervision | 0.01 | 0.38 | 0.39 | 0.01 | 0.49 | 0.50 | 0.00 | 0.09 | 0.09 | 0.00 | 0.09 | 0.09 |
|  | Service delivery | 1.01 | 0.90 | 1.91 | 1.10 | 0.78 | 1.88 | 0.52 | 0.58 | 1.10 | 0.52 | 0.71 | 1.23 |
|  | Record-keeping, monitoring and evaluation | 0.08 | 0.13 | 0.21 | 0.00 | 0.09 | 0.09 | 0.00 | 0.03 | 0.04 | 0.00 | 0.15 | 0.15 |
|  | Waste management | 0.09 | 0.04 | 0.13 | 0.19 | 0.00 | 0.19 | 0.01 | 0.01 | 0.02 | 0.01 | 0.00 | 0.01 |
|  | AEFI management | 0.00 | 0.00 | 0.00 | 0.00 | 0.01 | 0.01 | 0.00 | 0.00 | 0.00 | 0.00 | 0.00 | 0.00 |
|  | Microplanning and social mobilization | 0.00 | 0.03 | 0.03 | 0.00 | 0.03 | 0.03 | 0.00 | 0.00 | 0.00 | 0.00 | 0.10 | 0.10 |
| **Resource types** | Paid labor | 0.01 | 2.74 | 2.75 | 0.01 | 2.40 | 2.41 | 0.00 | 0.73 | 0.73 | 0.00 | 0.85 | 0.85 |
|  | Unpaid overtime | 0.00 | 0.06 | 0.06 | 0.00 | 0.05 | 0.05 | 0.00 | 0.05 | 0.05 | 0.00 | 0.09 | 0.09 |
|  | Volunteer labor | 0.00 | 0.01 | 0.01 | 0.00 | 0.00 | 0.00 | 0.00 | 0.04 | 0.04 | 0.00 | 0.23 | 0.23 |
|  | Per diems & travel allowances | 0.01 | 0.00 | 0.01 | 0.01 | 0.00 | 0.01 | 0.00 | 0.00 | 0.00 | 0.00 | 0.00 | 0.00 |
|  | Injection incentives | 0.73 | 0.00 | 0.73 | 0.84 | 0.00 | 0.84 | 0.25 | 0.00 | 0.25 | 0.25 | 0.00 | 0.25 |
|  | Immunization supplies | 0.20 | 0.00 | 0.20 | 0.19 | 0.00 | 0.19 | 0.20 | 0.00 | 0.20 | 0.20 | 0.00 | 0.20 |
|  | Stationery & other supplies | 0.07 | 0.00 | 0.07 | 0.07 | 0.00 | 0.07 | 0.07 | 0.00 | 0.07 | 0.07 | 0.00 | 0.07 |
|  | Transport fuel | 0.60 | 0.00 | 0.60 | 0.59 | 0.00 | 0.59 | 0.02 | 0.00 | 0.02 | 0.02 | 0.00 | 0.02 |
|  | Vehicle maintenance | 0.00 | 0.00 | 0.00 | 0.00 | 0.00 | 0.00 | 0.00 | 0.00 | 0.00 | 0.00 | 0.00 | 0.00 |
|  | Cold chain repair & energy | 0.00 | 0.15 | 0.15 | 0.00 | 0.04 | 0.04 | 0.00 | 0.00 | 0.00 | 0.00 | 0.00 | 0.00 |
|  | IEC & printing | 0.00 | 0.10 | 0.10 | 0.00 | 0.01 | 0.01 | 0.00 | 0.00 | 0.00 | 0.00 | 0.00 | 0.00 |
|  | Workshop & meetings | 0.03 | 0.00 | 0.03 | 0.05 | 0.00 | 0.05 | 0.00 | 0.00 | 0.00 | 0.00 | 0.00 | 0.00 |
|  | Others recurrent costs | 0.26 | 0.00 | 0.26 | 0.28 | 0.00 | 0.28 | 0.01 | 0.00 | 0.01 | 0.01 | 0.00 | 0.02 |
|  | Cold chain equipment | 0.14 | 0.14 | 0.28 | 0.04 | 0.25 | 0.29 | 0.00 | 0.00 | 0.01 | 0.00 | 0.01 | 0.01 |
|  | Vehicles | 0.00 | 0.03 | 0.03 | 0.00 | 0.03 | 0.03 | 0.00 | 0.00 | 0.00 | 0.00 | 0.00 | 0.00 |
|  | Incinerator | 0.00 | 0.00 | 0.00 | 0.00 | 0.00 | 0.00 | 0.00 | 0.00 | 0.00 | 0.00 | 0.00 | 0.00 |
| **OVERALL** | | **2.07** | **3.22** | **5.29** | **2.03** | **2.83** | **4.85** | **0.56** | **0.83** | **1.39** | **0.56** | **1.19** | **1.75** |

Table S6. Detailed characteristics of delivery sites (Low-volume period).

|  | Volume weighted average (rounded) | | | | | | |
| --- | --- | --- | --- | --- | --- | --- | --- |
|  | Overall | Facility-Based | Temporary sites | Hanoi | | | Dak Lak |
|  |  |  |  | Overall | Urban | Peri-Urban | Overall/ Remote |
| Number of sites | 16 | 13 | 3 | 15 | 5 | 10 | 1 |
| Vaccination team members per site | 8 | 8 | 7 | 8 | 10 | 6 | 8 |
| Vaccinators per site | 1 | 2 | 1 | 1 | 2 | 1 | 2 |
| Person-minute spent to deliver one dose per site | 50 | 47 | 61 | 49 | 91 | 26 | 100 |
| Doses delivered per day | 98 | 101 | 84 | 99 | 111 | 90 | 32 |

Table S7. Detailed characteristics of delivery sites (High-volume period).

|  | Volume weighted average (rounded) | | | | | | |
| --- | --- | --- | --- | --- | --- | --- | --- |
|  | Overall | Facility-Based | Temporary sites | Hanoi | | | Dak Lak |
|  |  |  |  | Overall | Urban | Peri-Urban | Overall/ Remote |
| Number of sites | 23 | 11 | 12 | 20 | 11 | 9 | 3 |
| Vaccination team members per site | 33 | 20 | 38 | 34 | 30 | 39 | 24 |
| Vaccinators per site | 5 | 5 | 6 | 6 | 8 | 3 | 2 |
| Person-minute spent to deliver one dose per site | 21 | 32 | 41 | 39 | 47 | 30 | 31 |
| Doses delivered per day | 412 | 271 | 467 | 417 | 414 | 421 | 347 |

Figure S1. Economic cost per dose across delivery period, by resource type.


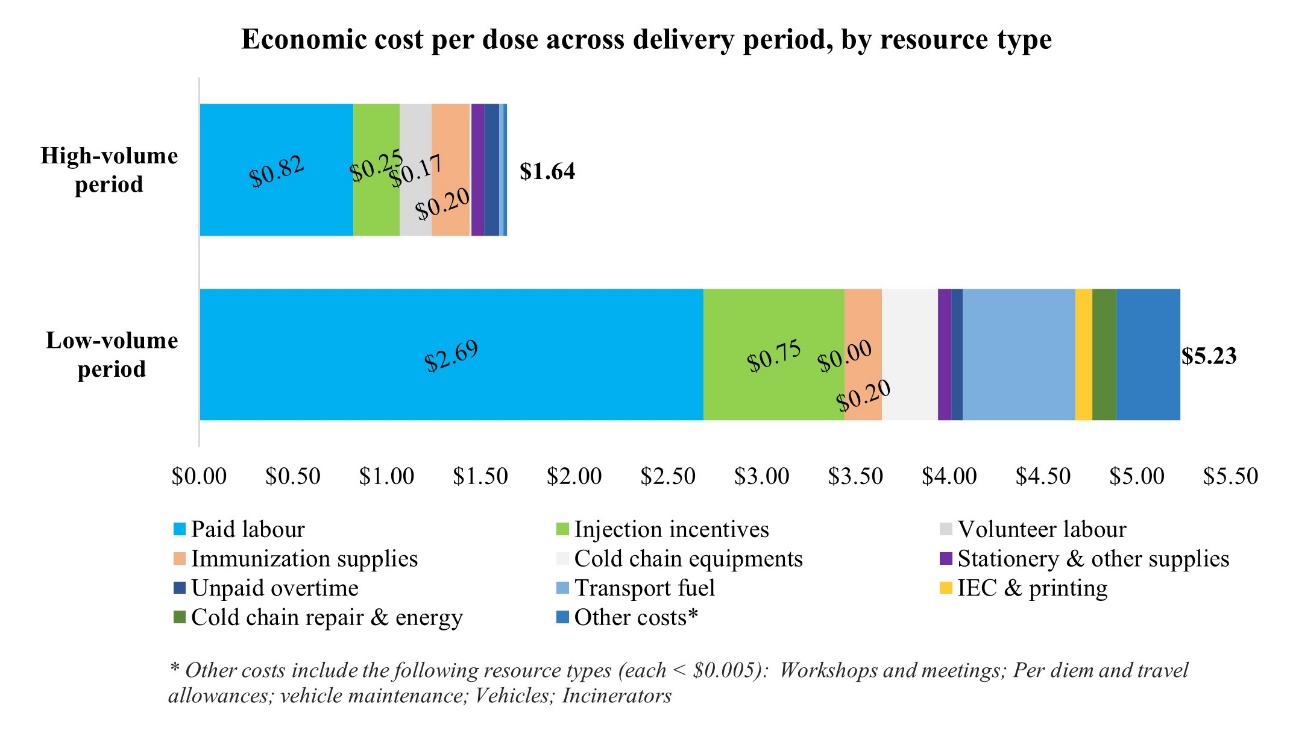


Figure S2. Economic cost per dose across geographic areas, by resource type.


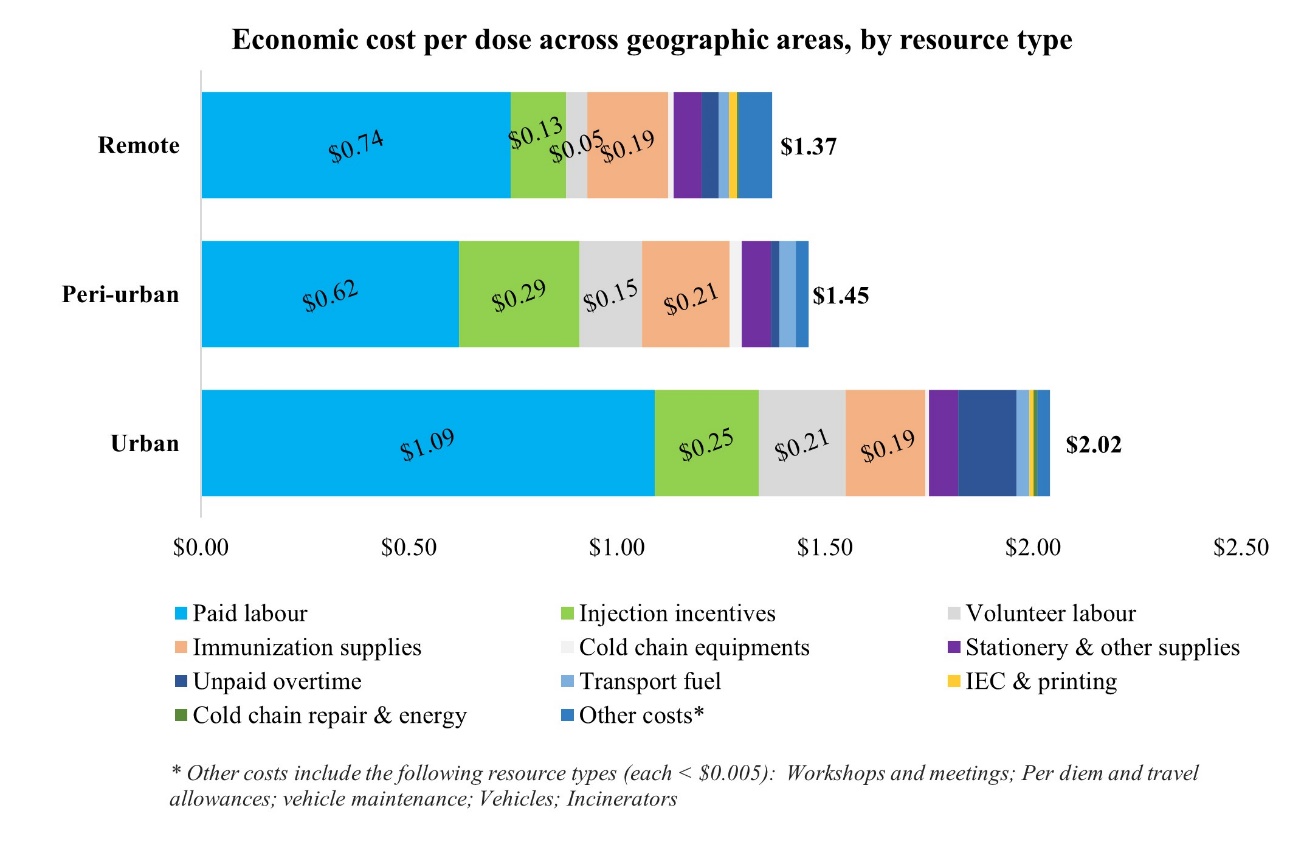


Figure S3. Economic cost per dose across provinces, by resource type.


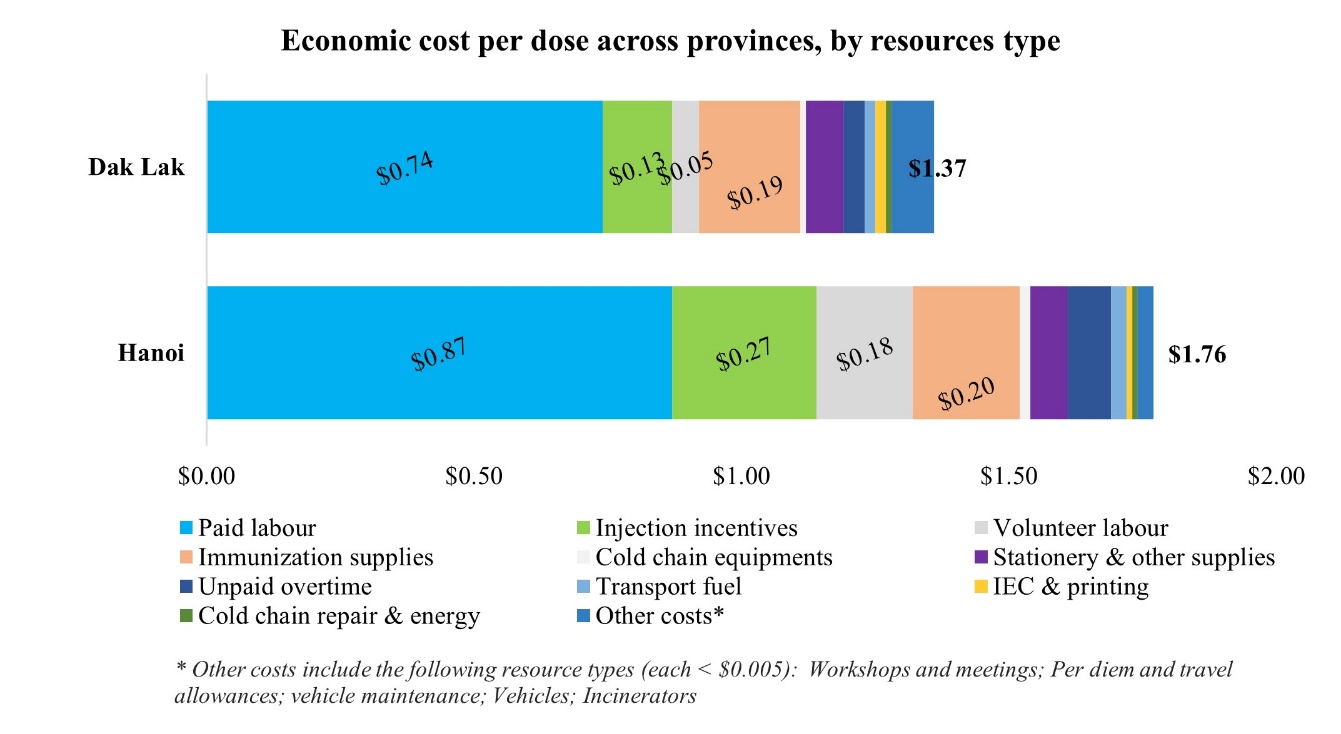


Figure S4. Economic cost per dose across strategies, by program activities.


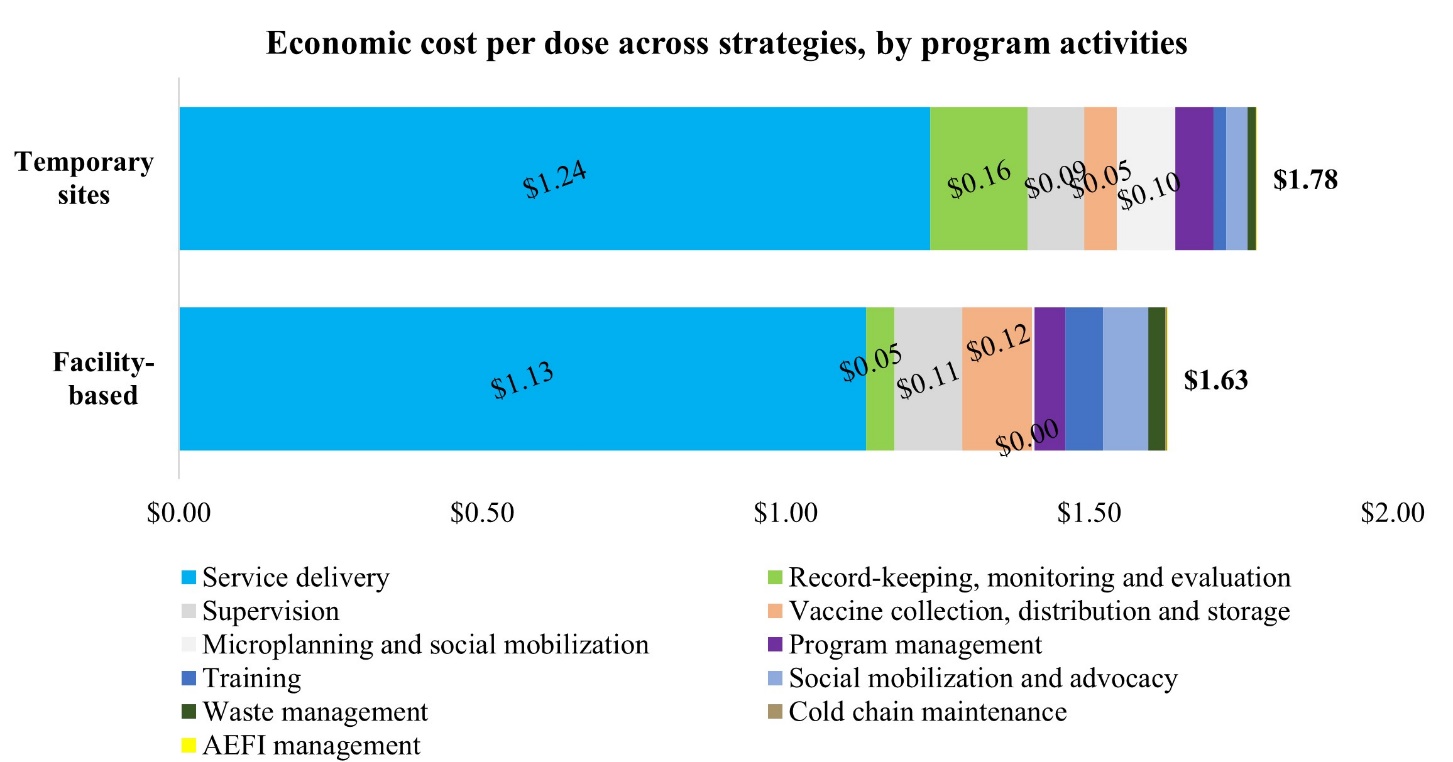


Figure S5. Economic cost per dose across provinces, by program activities.


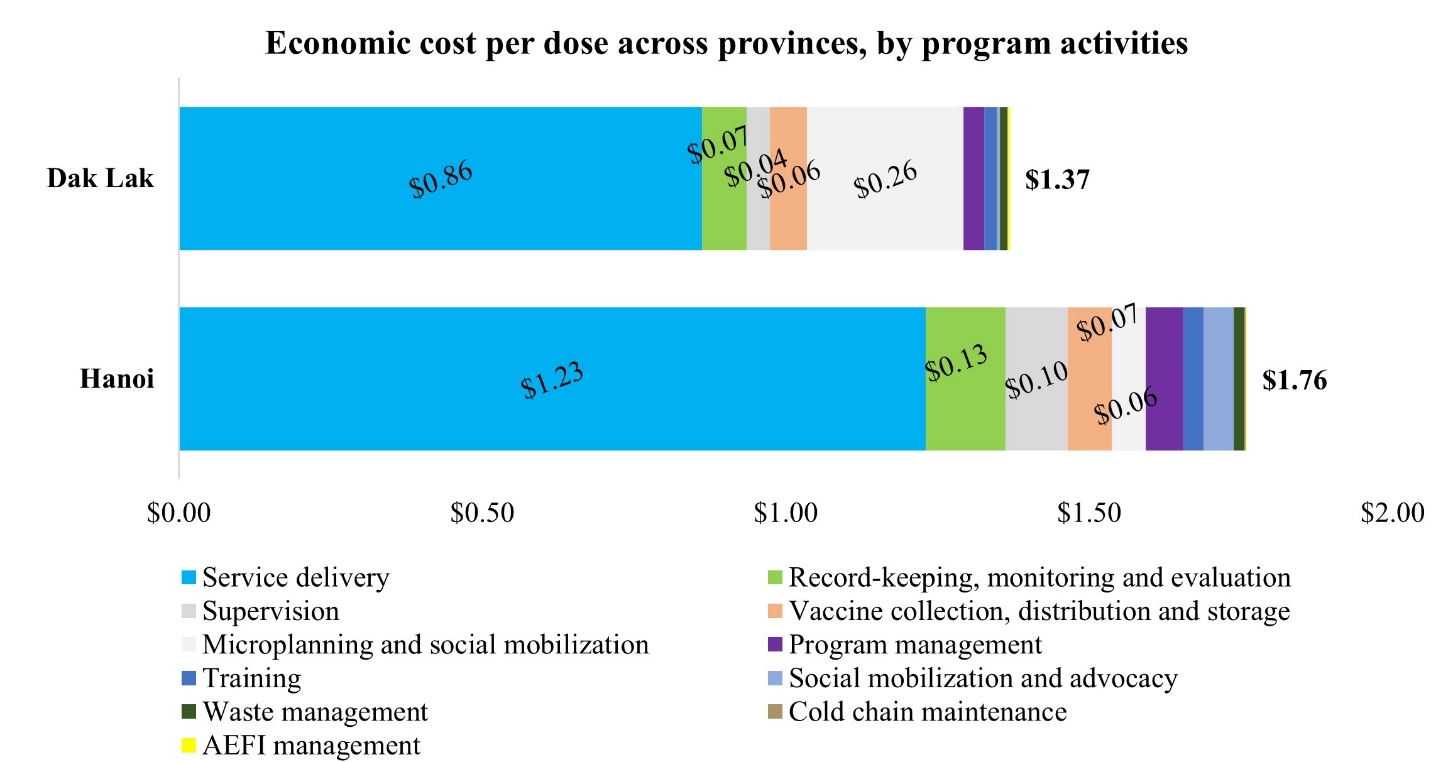


Figure S6. Economic cost per dose across geographic areas, by program activities.


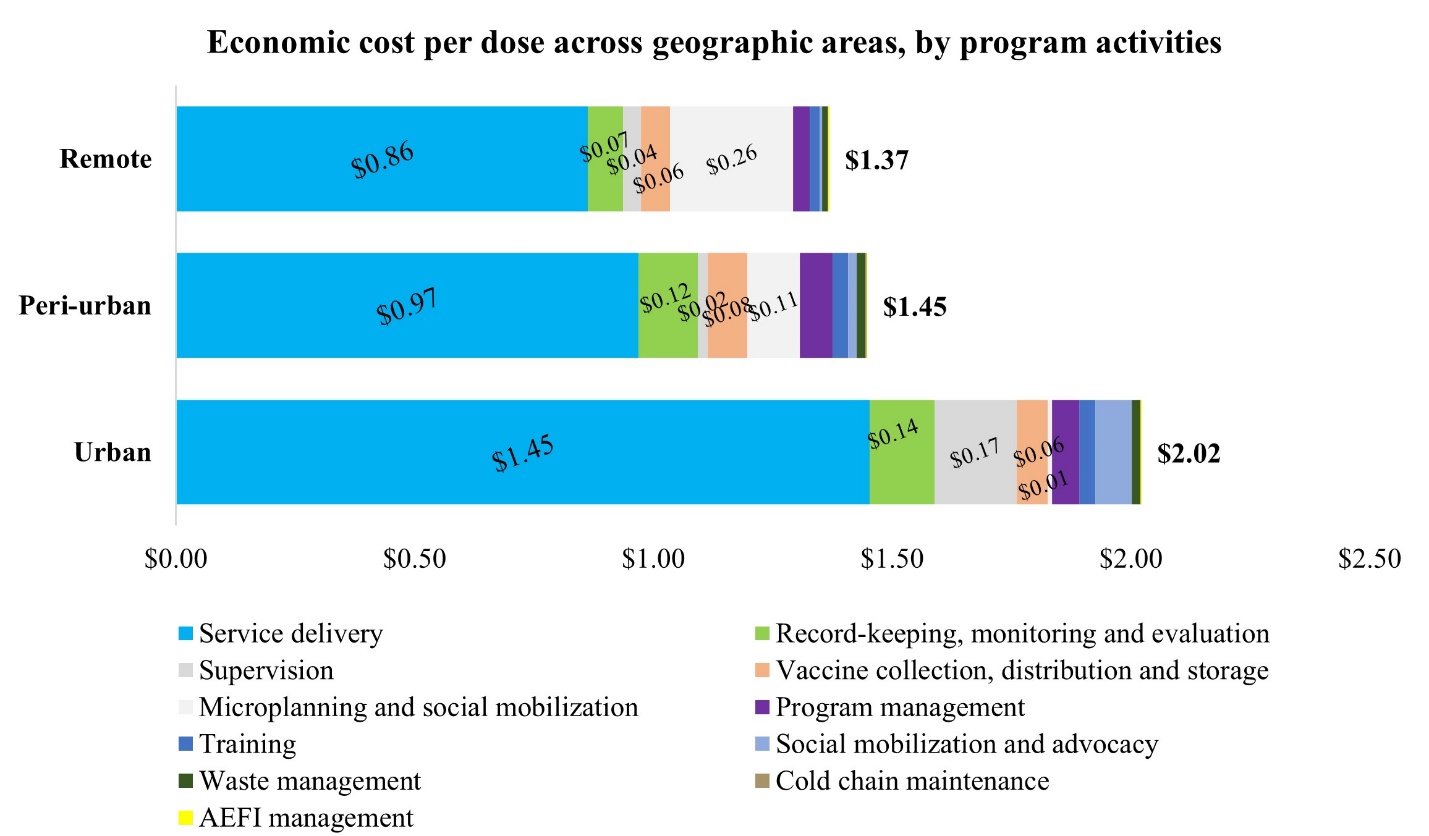

Supplement: Supplementary file 1 — Supplementary Material 1 [file 12913_2024_11202_MOESM1_ESM.docx]
